# Integrated Nicotine Replacement and Behavioral Support to Reduce Smoking in Opioid Agonist Therapy: A Randomized Clinical Trial

**DOI:** 10.1001/jamapsychiatry.2024.4801

**Published:** 2025-02-12

**Authors:** Karl Trygve Druckrey-Fiskaaen, Tesfaye Madebo, Jan Tore Daltveit, Jørn Henrik Vold, Einar Furulund, Fatemeh Chalabianloo, Torgeir Gilje Lid, Lars Thore Fadnes

**Affiliations:** 1 Bergen Addiction Research, Department of Addiction Medicine, Haukeland University Hospital, Bergen, Norway; 2 Department of Global Public Health and Primary Care, University of Bergen, Bergen, Norway; 3 Norwegian Research Center for Agonist Treatment of Substance Use Disorders, Department of Addiction Medicine, Haukeland University Hospital, Bergen, Norway; 4 Department of Respiratory Medicine, Stavanger University Hospital, Stavanger, Norway; 5 Department of Clinical Science, University of Bergen, Bergen, Norway; 6 Division of Psychiatry, Haukeland University Hospital, Bergen, Norway; 7 Centre for Alcohol and Drug Research, Stavanger University Hospital, Stavanger, Norway; 8 Oral Health Centre of Expertise Rogaland, Stavanger, Norway; 9 Department of Public Health, University of Stavanger, Stavanger, Norway

## Abstract

**Importance:**

Approximately 85% of individuals receiving opioid agonist therapy for opioid dependence smoke tobacco. Despite the significant health risks associated with smoking-related diseases, there has been limited evaluation of smoking interventions tailored to this population.

**Objective:**

To determine the effectiveness of an intervention combining nicotine replacement with brief behavioral support in reducing cigarette use.

**Design, Setting, and Participants:**

This multicenter randomized clinical trial was conducted from April 2022 to October 2023 in 7 specialized opioid agonist therapy clinics in Bergen and Stavanger, Norway. The analyst was blinded to patient groupings. Assessors (study nurses) were not fully blinded to participant allocation. Individuals diagnosed with opioid dependency receiving opioid agonist therapy at participating clinics and smoking at least 1 cigarette per day were eligible for participation. Data analysis was performed from December 2023 through October 2024.

**Intervention:**

In addition to standard opioid agonist therapy, participants in the intervention group received a 16-week integrated treatment combining nicotine replacement with brief behavioral support. Participants in the control group received only standard opioid agonist therapy.

**Main Outcomes and Measures:**

The primary outcome was at least a 50% reduction in the number of cigarettes smoked, self-reported as cigarette use in the past 7 days at week 16. The analysis followed intention-to-treat principles. Cigarette use was self-reported as per the timeline-follow-back method.

**Results:**

Among the 259 participants (mean [SD] age, 48.5 [10.4] years; 80 [30.9%] female), 135 were allocated to the intervention group and 124 to the control group. The odds ratio of at least halving the number of cigarettes smoked was 2.07 (95% CI, 1.14-3.75) in the intervention group compared with the control group.

**Conclusions and Relevance:**

Providing integrated nicotine replacement and behavioral support at opioid agonist treatment clinics effectively helped opioid-dependent participants reduce the number of cigarettes smoked.

**Trial Registration:**

ClinicalTrials.gov Identifier: NCT05290025

## Key Points

**Question:**

Can an integrated intervention combining nicotine replacement and brief behavioral support reduce the number of cigarettes smoked among patients receiving opioid agonist therapy?

**Findings:**

In this randomized clinical trial including 259 participants, twice as many participants in the intervention group halved the number of cigarettes smoked compared with the control group, a significant difference.

**Meaning:**

The findings suggest that providing nicotine replacement and brief behavioral support alongside opioid agonist therapy at clinics is effective in helping patients reduce the number of cigarettes smoked.

## Teaser

This randomized clinical trial evaluates the effectiveness of an intervention combining nicotine replacement with brief behavioral support in reducing cigarette use among adults with opioid dependency in Norway.

## Introduction

Approximately 85% of individuals diagnosed with opioid dependence smoke tobacco.^1^ Notably, quit rates among those receiving opioid agonist therapy (OAT) for opioid dependence are significantly lower compared with the general population.^2,3,4,5^ Several factors contribute to the low efficacy of smoking cessation interventions within the OAT population.^6,7^ Nicotine amplifies opioid reward, enhances the acute antinociceptive effects of opioids, and ameliorates opioid withdrawal symptoms.^6^ Opioid-dependent smokers are more nicotine dependent than smokers without opioid dependence.^8^ The rewarding effects of acute nicotine analgesia and smoking-related increase in long-term pain may explain why smokers have an increased risk of chronic pain and use opioids more frequently than nonsmokers.^9,10,11^ There is limited access to smoking cessation services within OAT programs, where the primary focus is often on treating opioid dependence.^12^ Additionally, social norms and peer influence within the OAT community can reinforce smoking behavior, which is further linked to the high rates of co-occurring mental health disorders, such as anxiety, depression, and psychosis.^13,14,15,16,17^ Despite challenges related to nicotine and opioid codependence, many individuals undergoing OAT have reported motivation to change smoking habits.^13,18,19^ Interventions designed explicitly for smokers receiving OAT are therefore necessary to reduce smoking. This could improve overall health and narrow the health disparity between people with opioid dependence and the general population.

The high smoking rate significantly exacerbates the already concerning morbidity and mortality among individuals with opioid dependence. Smoking-related illnesses contribute to approximately 50% of the morbidity in this population.^20,21,22,23^ In populations of opioid-dependent individuals, around 30% present with chronic obstructive pulmonary disease (COPD), and 63% of patients undergoing OAT exhibited pulmonary pathologies in autopsy samples.^23,24,25^ Smokers who reduced the amount of tobacco smoked had lower risks of adverse health outcomes, such as peripheral arterial disease, lung cancer, COPD, and laryngeal cancer.^26,27^ Reducing tobacco-related morbidity and mortality, especially among opioid-dependent individuals with underlying smoking-related somatic disorders, through early interventions to reduce or quit smoking could significantly enhance health outcomes within these populations.^28^

Smoking cessation interventions, incorporating nicotine replacement therapy (NRT) alongside behavioral interventions or a combination of both, have shown increased rates of smoking abstinence, with no adverse impact on other substance use treatment outcomes.^29^ However, among individuals with opioid dependency, smoking cessation medications demonstrate reduced efficacy, offering only modest effectiveness in aiding tobacco cessation.^7,29,30^ Additional behavioral support appears to increase the chances of success, but behavioral support alone did not impact smoking behavior in opioid-dependent individuals.^7,30^ In the general population, reviews found that smoking reduction interventions supported by NRT increased the chance of quitting compared with reduction alone.^31^ Similarly, NRT significantly increased the risk ratio of at least halving the number of cigarettes smoked per day.^32,33,34^ Studies are needed to test whether similar effects can be observed in populations of opioid-dependent persons.

The objective of this randomized clinical trial was to investigate the effectiveness of an integrated 16-week smoking reduction intervention in reducing the number of cigarettes smoked per day for persons receiving opioid agonist therapy. The intervention combined nicotine replacement and behavioral support and was compared with standard opioid agonist therapy.

## Methods

### Study Design and Setting

We conducted a pragmatic, multicenter, individually randomized clinical superiority trial from April 2022 to October 2023 in 7 OAT clinics in Bergen and Stavanger, Norway. The trial protocol is available in Supplement 1. The target population was persons diagnosed with opioid dependence syndrome, according to the *International Statistical Classification of Diseases and Related Health Problems, Tenth Revision*, receiving OAT (eMethods 1 in Supplement 2).^35^ The South-Eastern Regional Ethical Committee reviewed and approved this study. This study followed the Consolidated Standards of Reporting Trials (CONSORT) reporting guidelines for randomized clinical trials. Study nurses obtained written informed consent after assessment for eligibility. Data analysis was performed from December 2023 through October 2024.

### Participants

Participants were recruited regardless of motivation to change smoking habits. Eligibility criteria were defined as (1) receiving OAT medication at least weekly and (2) smoking at least 1 cigarette (including tobacco mixed with cannabis) per day or 7 cigarettes per week for the past week. Participants were excluded if they (1) had allergies or prior anaphylactic reactions to the medication used, (2) smoked less than 3 times per week, or (3) used smoking cessation medications at inclusion. A total of 460 persons were assessed for eligibility. Following screening, 266 participants were randomized. See the Figure and eMethods 1 in Supplement 2 for details on recruitment, allocation, and analysis.

**Figure.**
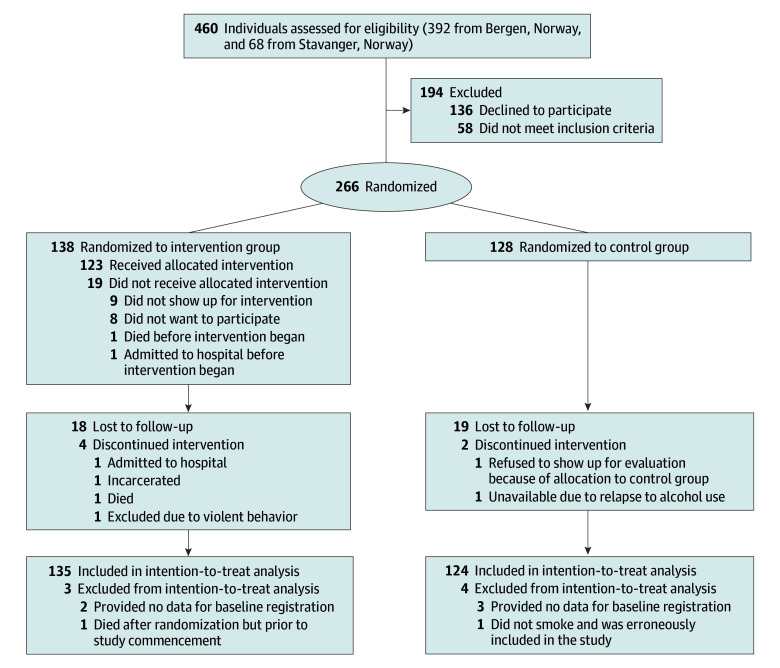
CONSORT Flow Diagram

### Randomization and Masking

We randomized participants by site, with a ratio of 1:1 using computer-generated blocks of 8, resulting in the assignment of 138 persons to the intervention group and 128 persons to the control group. Once a participant fulfilled all the eligibility criteria and signed the informed consent form, a unique participant identifier number was entered into the randomization spreadsheet, which generated the allocation to the study group. Complete blinding was considered difficult due to the pragmatic character of the trial and would have reduced external validity.^36^ Participants were informed of the key elements of the follow-up and evaluations but not of other follow-up alternatives used or the exact hypotheses for the study before consenting to participation. The analyst was blinded, while outcome assessors had access to randomization information but were asked to avoid noting this.

### Interventions

Study nurses completed identical assessments for participants in both study groups at trial visits at baseline and 16 weeks. Participants randomized to the control group received standard OAT and no NRT or behavioral support (eMethods 1 in Supplement 2).

Participants in the intervention group were offered a 16-week integrated smoking reduction intervention combining both brief behavioral support and prescription-free and free-of-charge NRT products. We used the term *integrated* to specify that the intervention was provided alongside the standard OAT by the staff at the clinic as an integral part of the care received at the OAT clinics. The behavioral support was inspired by motivational interviewing and psychoeducational techniques. It consisted of a short motivational talk covering the topics of smoking during the past week of the intervention, setting a goal for the intervention, and how to reduce or quit smoking. Participants were offered 16 weekly intervention sessions in which the behavioral support was delivered, cigarette use for the past day was recorded, and participants were provided with NRT products for the next week. Participant engagement was flexible (eMethods 1 in Supplement 2). Available medications were nicotine patches (7-21 mg/24 hours) and nicotine lozenges or chewing gum (in 1- or 2-mg units). Dosing of the NRT products followed the manufacturers’ instructions and was based on the participants’ reported cigarette use at baseline. The intervention was detailed in a published protocol and followed CONSORT reporting standards.^37,38^

### Outcomes and Assessments

We defined the primary outcome as achieving at least a 50% reduction in the number of cigarettes smoked by week 16 of the intervention period (range, 12-16 weeks after intervention initiation) compared with baseline. Cigarette use at baseline and evaluation (week 16) was estimated using timeline follow-back for the past 7 days.^39^ The amount of tobacco (as a fraction of a cigarette) smoked with cannabis was added to the number of cigarettes smoked (eMethods 3 in Supplement 2). Carbon monoxide levels in the exhaled air verified the self-reported cigarette use at baseline and 16 weeks. We defined individuals with a carbon monoxide concentration below 6 parts per million as nonsmokers.^40^

### Statistical Analysis

Sample size calculations estimated that if the intervention would reduce the number of cigarettes smoked by participants in the intervention group by 30%, 266 participants, equally distributed between the intervention and control groups, would be needed. However, it was later considered that a 50% reduction in smoking was required to be sufficiently clinically relevant. Thus, our primary analyses were changed accordingly, and an updated analysis plan was published prior to the completion of the study.^41^ A post hoc analysis with 90% power estimated that 123 participants per group (246 in total) would be needed (eMethods 2 in Supplement 2).

We used intention-to-treat principles when analyzing the data by including all patients who were randomized. Data missing at evaluation were set equal to baseline values. For the per-protocol analysis, we excluded participants allocated to the intervention group who had not completed at least 50% of the intervention sessions. All tests were 2-sided. The threshold for statistical significance was set to *P* < .05. Categorical variables were summarized as percentages or medians with interquartile range. Continuous variables were presented as means with standard deviation. The outcomes were assessed to ensure that the assumptions of independent outcomes, limited influence of outliers, and nonmulticollinearity held. We estimated the absolute pre vs post differences, including 95% CIs, between the study groups for the number of smokers, number of cigarettes smoked, and patients with carbon monoxide levels less than 6 parts per million. Logistic regression models were built to test for potential differences in smoking reduction between the study groups. We performed sensitivity analyses evaluating the impact of excluding participants with missing data in the outcome variable from the intention-to-treat analysis and using the median number of intervention sessions for per-protocol analysis. A sensitivity analysis of the logistic regression model was built adjusting for potential confounders if randomization did not fully succeed to minimize these (age group, sex, OAT medication, injection of mixture or tablets, pack-years, COPD^42^). Any missing data in the confounder variables were estimated using multiple imputation.

Hypothesis-generating subgroup analyses of the primary outcome were performed, running the logistic regression model stratified by the following subgroups: age groups, sex, spirometry results, OAT medication, injection of mixture or tablets, pack-years, and years of smoking.

## Results

Among the 266 participants, data were available for 259 participants. Of them, 80 (30.9%) were female; the mean (SD) age was 48.5 (10.4) years (range, 20.6-70.8 years) (Table 1). Participants reported smoking a mean (SD) of 20.4 (15.7) pack-years and 11.7 (8.1) cigarettes per day at baseline. Buprenorphine was the OAT medication most frequently used (137 of 259 participants [52.9%]). Basic characteristics of the per-protocol population are provided in eTable 1 in Supplement 2. Participants’ goals for the trial and ratings of self-confidence are shown in eTables 2 through 4 in Supplement 2.

**Table 1.** Demographic Characteristics of Study Participants in the Intention-to-Treat Analysis

Characteristic	Intervention (n = 135)	Control (n = 124)
Sex, No. (%)		
Female	42 (31.1)	38 (30.6)
Male	93 (68.9)	86 (69.4)
Age, y		
Mean (SD) [range]	47.3 (10.4) [24.0-69.4]	49.8 (10.4) [20.6-70.8]
By group, No. (%)		
<40	34 (25.2)	22 (17.7)
40-60	84 (62.2)	83 (66.9)
>60	17 (12.6)	19 (15.3)
BMI, mean (SD)	25.5 (5.1)	26.2 (6.6)
OAT medication, No. (%)^a^		
Methadone and others^b^	63 (46.7)	59 (47.6)
Buprenorphine	72 (53.3)	65 (52.4)
Stable living conditions, No. (%)^c^	126 (94.0)	117 (94.4)
Education, No. (%)		
Not finished basic education^d^	4 (3.1)	7 (5.8)
Finished basic education^d^	67 (51.5)	56 (46.7)
High school or higher^e^	59 (45.4)	57 (46.0)
Social benefits as income, No. (%)	133 (99.3)	123 (99.2)
Substance use, No. (%)^f^		
Opioids^g^		
None	102 (75.6)	99 (80.5)
≤3 Times/wk	23 (17.0)	20 (16.3)
>3 Times/wk	10 (7.4)	4 (3.2)
Alcohol		
None	64 (47.4)	57 (46.3)
≤3 Times/wk	54 (40.0)	51 (41.5)
>3 Times/wk	17 (12.6)	15 (12.2)
Stimulants^h^		
None	85 (63.0)	85 (69.1)
≤3 Times/wk	34 (25.2)	25 (20.3)
>3 Times/wk	16 (11.9)	13 (10.6)
Benzodiazepines		
None	58 (43.0)	64 (52.0)
≤3 Times/wk	44 (32.6)	28 (22.8)
>3 Times/wk	33 (24.4)	31 (25.1)
Cannabis		
None	49 (36.3)	31 (25.2)
≤3 Times/wk	26 (19.3)	35 (28.5)
>3 Times/wk	60 (45.1)	57 (46.4)
Daily tobacco use, No. (%)	127 (94.1)	119 (96.7)
Smoking debut age, mean (SD), y	14.3 (3.4)	14.1 (3.5)
>15 y of smoking, No. (%)	129 (96.3)	117 (94.4)
Pack-years, mean (SD)	21.0 (15.9)	19.7 (15.4)
No. of cigarettes/d, mean (SD)	12.5 (8.3)	10.8 (7.8)
Carbon monoxide, mean (SD), ppm	16.0 (8.6)	15.1 (8.5)
Probable COPD, No. (%)^i^	24 (21.8)	37 (37.4)
Injection of tablets or mixture, No. (%)	20 (15.9)	15 (12.7)
Injection frequency, No. (%)		
≤3 Times/wk	16 (80.0)	10 (66.7)
>3 Times/wk	4 (20.0)	5 (33.3)

Abbreviations: BMI, body mass index (calculated as weight in kilograms divided by height in meters squared); COPD, chronic obstructive pulmonary disease; OAT, opioid agonist therapy; ppm, parts per million.

^a^ Medication being used at baseline.

^b^ Others include morphine sulfate formulations in 11 participants (4.2%).

^c^ Living in an owned or rented home or being incarcerated.

^d^ In Norway, the first 10 school years are mandatory for all pupils.

^e^ Grades 11 through 13.

^f^ Self-reported frequency of substance use in the last 30 days prior to baseline assessment.

^g^ Illegal opioids not part of the OAT program.

^h^ Amphetamines and cocaine.

^i^ At least 1 spirometry result indicating a forced expiratory volume in the first second of expiration to forced vital capacity ratio below the lower limit of normality.^42^

Of the 259 participants, 135 were randomized to the intervention group and 124 to the control group. In total, 216 (83.4%) of the study sample completed the 16-week evaluation. In the intervention group, 37 participants completed no intervention sessions (mode), the median number of sessions attended was 7 (range, 0-16 sessions), and 51 of 135 participants (37.8%) completed at least 50% of the intervention sessions (eFigure 1 and eTable 5 in Supplement 2). The timing of the dropouts is presented in eFigure 2 in Supplement 2.

In the intervention group, 40 of 135 patients (29.6%) at least halved the number of cigarettes smoked per day compared with 21 of 124 patients (16.9%) in the control group (odds ratio [OR], 2.07 [95% CI, 1.14-3.75]; adjusted OR, 1.82 [95% CI, 0.97-3.40]) (Table 2; eTable 6 in Supplement 2). No significant differences existed in the number of participants reporting smoking cessation between the study groups. Missing data did not impact the results (OR, 2.14 [95% CI, 1.16-3.96]) (eTables 7 and 8 in Supplement 2).

**Table 2.** Effect of Intervention on Smoking Behavior

Outcome	Events, No./total No. (%)		Absolute difference, % (95% CI)	Logistic regression			
				Unadjusted		Adjusted^a^	
	Intervention	Control		OR (95% CI)	*P* value	OR (95% CI)	*P* value
≥50% Reduction in No. of cigarettes at 16 wk							
ITT^b^	40/135 (29.6)	21/124 (16.9)	−12.7 (−23.0 to −2.4)	2.07 (1.14-3.75)	.02	1.82 (0.97-3.40)	.06
PP^c^^,^^d^	23/51 (45.1)	21/103 (20.4)	−24.7 (−39.6 to −9.8)	3.21 (1.54-6.66)	.002	3.12 (1.41-6.87)	.005
PP, median^d^^,^^e^	29/64 (45.3)	21/103 (20.4)	24.9 (11.0 to 38.9)	3.24 (1.63-6.43)	.001	3.36 (1.64-6.87)	.001
Smokers at 16 wk							
ITT^b^^,^^f^	134/135 (99.3)	119/124 (96.0)	3.3 (−0.4 to 7.0)	0.17 (0.02-1.54)	.12	0.22 (0.02-2.64)	.23
PP^d^	51/51 (100)	98/103 (95.1)	4.8 (−1.1 to 10.8)	NA	NA	NA	NA
Carbon monoxide^g^							
<6 ppm	17/134 (12.7)	14/122 (11.5)	−1.2 (−9.3 to 6.9)	1.12 (0.53-2.38)	.77	NA	NA
At 16 wk, mean (SD), ppm	15.74 (9.53)	15.90 (9.23)	−0.16 (−2.48 to 2.15)^h^	NA	NA	NA	NA
Cigarettes smoked at 16 wk, mean (SD), No./d, ITT^b^	8.5 (6.0)	9.7 (8.2)	1.2 (−0.5 to 3.0)^i^	NA	NA	NA	NA
SAE, assumed linked	0	0	NA	NA	NA	NA	NA

Abbreviations: ITT, intention to treat; NA, not applicable; OR, odds ratio; PP, per protocol; ppm, parts per million; SAE, severe adverse event.

^a^ Adjusted for age group, sex, opioid agonist therapy medication, injection of mixture or tablets, pack-years, chronic obstructive pulmonary disease, and cannabis smoking at baseline. Missing exposure values were imputed, in total 67 imputations for ITT analysis and 25 for PP analysis.

^b^ Participants were assessed according to randomization regardless of adherence to the trial. If data on the primary outcome were missing at 16 weeks, the results were set equal to baseline.

^c^ All participants who completed at least 50% of the intervention sessions.

^d^ If data on the primary outcome were missing at 16 weeks, the person was excluded from the analysis (complete case).

^e^ Per-protocol threshold was set at the median number (7) of intervention sessions attended.

^f^ A person smoking at least 1 cigarette per day or 7 cigarettes per week.

^g^ Carbon monoxide levels in the exhaled air.

^h^ Expressed as carbon monoxide level in parts per million.

^i^ Expressed as number of cigarettes smoked per day.

The per-protocol analyses showed that 23 of 51 participants (45.1%) in the intervention group at least halved the daily number of cigarettes smoked, compared with 21 of 103 participants (20.4%) in the control group (OR, 3.21 [95% CI, 1.54-6.66]). Changing the per-protocol threshold had little impact on the results (Table 2).

Subgroup analysis of the primary outcome indicated that the effect of the intervention was stronger among men, participants aged 40 to 60 years, those receiving buprenorphine, not injecting, and smoking for more than 15 years and more intensely (eTables 9 and 10 in Supplement 2).

The Spearman ρ between the carbon monoxide levels and the number of cigarettes smoked at week 16 was 0.3897 (*P* < .001) (eFigure 3 in Supplement 2). Correlation was similar in the intervention and control groups (eMethods 4 in the Supplement 2).

## Discussion

This study showed that an integrated smoking reduction intervention with NRT and behavioral support was twice as effective as standard treatment in reducing the number of cigarettes smoked by at least 50%. To our knowledge, no other studies have evaluated the effect of integrated NRT and behavioral support among patients receiving OAT. Our intervention has parallels to OAT, which incorporates agonist therapy combined with psychosocial support in a harm reduction perspective, while smoking interventions typically have an expressed quitting focus, which is more parallel to the focus on abstinence. Our results, with many participants slowly reducing smoking without achieving cessation within the trial period, could also indicate the usefulness of a longer duration.

As sustained smoking cessation has been notoriously difficult to achieve for opioid-dependent persons, integrated interventions like ours could provide an efficient means to reduce smoking-associated health risks and help patients receiving OAT quit smoking.^26,27,43,44,45^ In the general population, smoking reduction interventions supported by NRT increased the chance of quitting compared with reduction alone and increased the risk ratio of at least halving the number of cigarettes smoked per day.^31,32,33,34^ Our study showed that this is also applicable to persons receiving OAT, regardless of motivation to change smoking habits. Low motivation and concern about symptom management were barriers to smoking cessation among persons with mental illnesses, including substance use.^46^ A sole expectation of smoking cessation without the possibility of reducing could be perceived as unattainable and thus reduce readiness to change, impacting future quit attempts.^47^

The small difference in mean number of cigarettes smoked at the end indicates that the current duration was unlikely sufficient to help all participants reduce smoking substantially. Among individuals with serious mental illness, clinician education and the engagement of community health workers significantly increased smoking cessation rates.^48,49^ In the general population, an average of 6 quit attempts are needed to achieve long-term abstinence.^50^ Making NRT and behavioral support accessible as an integral part of OAT with additional long-term community health support could aid opioid-dependent persons in making several quit attempts, eventually achieving smoking cessation.

In our study, nearly 40% of the participants in the intervention group completed at least half of the intervention sessions. In clinical trials, low adherence to NRT has been associated with reduced effectiveness.^51^ Life events, substance use and craving, and anxiety have been identified as barriers to participation among substance-dependent persons and other disadvantaged groups.^46,52,53^ These barriers likely explain the low participation in our trial. Factors primarily impacting adherence to NRT were related to conscious decision-making, nicotine dependence, and mental health status.^54^ Assessing how these barriers and facilitators apply to patients receiving OAT may be a potential means of increasing participation in further studies.

The present study showed that the number of patients who reported stopping smoking was substantially lower than those whose measured carbon monoxide level was below the cutoff for smoking cessation. A meta-analysis of self-reported and biochemically verified smoking cessation estimated that 47% of persons who self-reported smoking cessation could be biochemically verified as abstinent.^55^ Carbon monoxide has a half-life of 4.5 hours, and baseline carbon monoxide levels vary between individuals.^56^ Thus, the discrepancy in measurement and self-report may indicate that participants had varying smoking intensity throughout the day. As we included the tobacco proportion mixed with cannabis in the cigarette count, a failure to report smoking cessation may reflect continued cannabis use.

Subgroup analysis indicated that women less often reduced smoking than men, which corresponds with other studies in the general population.^57^ Fear of weight gain and negative mood seem to predict lower success of smoking cessation among women.^58^ The odds of reducing cigarette use was lower among participants receiving methadone than buprenorphine. Nicotine and methadone share effects such as increased drug liking and euphoria.^59^ Nicotine and methadone could attenuate the withdrawal symptoms of the other drug.^6,59^ The partial agonist nature of buprenorphine possibly results in a lower risk of pharmacological interaction between nicotine and buprenorphine, easing the reduction of cigarettes smoked.^60^ Participants who did not report injecting had higher odds of reducing smoking, indicating that a more stable life situation could impact smoking behavior. In our study, participants with longer and heavier smoking had higher odds of reducing cigarettes. In the general population, a higher degree of smoking dependence reduces the chance of quitting, whereas older age is associated with smoking cessation.^61,62^ Our results are probably impacted by the fact that 95% of participants reported smoking longer than 15 years.

### Strengths and Limitations

Strengths of this study were that it used a trial design and included all eligible persons willing to participate regardless of motivation to change smoking habits. It tested a smoking reduction intervention integrated into the regular treatment at OAT outpatient clinics. Previous studies have indicated that providing smoking cessation intervention, regardless of motivation to quit, increases motivation to quit and reduces the number of cigarettes smoked per day.^43,44,63,64^ Our study sample was comparable to the Norwegian sample of persons receiving OAT in terms of sex (30.9% vs 30.4% female, respectively) and mean age (48.5 vs 48.1 years), indicating generalizability to a broader population receiving OAT.^65^ Limitations were the inclusion of the tobacco fraction mixed with cannabis in the number of cigarettes recorded, which made exact effect evaluation on tobacco smoking difficult and may have resulted in fewer participants reporting smoking cessation. The primary objective was slightly modified from the initial protocol. The change was described in the updated statistical plan published prior to the completion of the study.^41^ This resulted in the study having slightly higher power than planned. Block randomization and stratification by site were used to reduce the effect of potential confounders, yet some variables, such as COPD, were slightly unevenly distributed. Our study was not designed and powered to adjust for several variables; thus, the confidence intervals of the adjusted analyses should be interpreted with caution. Research nurses occasionally provided guidance to clinicians on how to deliver the intervention. Thus, the assessor blinding was partly compromised, which could introduce a social desirability or expectation bias. However, we consider substantial biases unlikely. Our study did not investigate the effects of behavioral support or NRT separately, but as a combined intervention. However, the effect size in our study could guide potential future studies looking into separate components. We initially planned to offer varenicline as an option for NRT. Due to varenicline being withdrawn from the European market by the producer, this product was not available for our trial.^66^ This study reports the results of being offered an intervention but does not evaluate the sustainability of the results.

## Conclusions

This randomized clinical trial of an integrated smoking reduction intervention, provided alongside ordinary OAT, demonstrated that being offered a combination of NRT and brief behavioral support reduced smoking, even though few managed to stop smoking during the intervention. The results support making smoking reduction interventions available as a standardized and integrated part of OAT, providing opioid-dependent persons with repeated opportunities to quit or reduce smoking. System changes allowing for continued and free-of-charge smoking reduction and cessation treatment could have the potential to reduce smoking-related morbidity and mortality among patients receiving OAT.

## Notes

Supplement 1. Trial Protocol

Supplement 2. **eMethods 1.** Details on Setting, Participants, and Interventions **eMethods 2.** Statistical Details **eMethods 3.** Details on Outcome and Measures **eMethods 4.** Sensitivity Analysis of Correlation of Self-Reported Cigarettes and Carbon Monoxide **eTable 1.** Demographics of Per Protocol Sample **eTable 2.** Participants’ Goals at the Start of the Trial (n = 135) **eTable 3.** Participants’ Estimation of the Time Needed to Achieve Smoking Reduction/Cessation Goal (n = 135) **eTable 4.** Participants’ Self-Reported Confidence in Success and Rating of Importance of Intervention at Baseline (n = 135) **eTable 5.** Overview of Study Visits Attended for Persons in the Intervention Group (n = 135) **eTable 6.** Adjusted Intention to Treat Analysis of Primary Outcome, With Imputation of Missing Variables (n = 259) **eTable 7.** Effect on Primary Outcome of Excluding Persons With Missing Values in the Primary Outcome **eTable 8.** Adjusted Intention to Treat Analysis of Primary Outcome, Without Imputation of Missing Variables (n = 200) **eTable 9.** Subgroup Analysis of the Primary Outcome Stratified by Baseline Variables **eTable 10.** Subgroup Analysis Assessing the Differential Effect of Various Subgroups on Primary Outcome (n = 259) **eFigure 1.** The Percentage of Study Visits Attended for Participants in the Intervention Arm **eFigure 2.** Timing of Drop-Out for Participants in the Intervention Arm **eFigure 3.** Scatter Plot of Self-Reported Number of Cigarettes and Carbon Monoxide Measurement
eReferences

Supplement 3. Data Sharing Statement
