# Supplementary material for: Integrated Nicotine Replacement and Behavioral Support to Reduce Smoking in Opioid Agonist Therapy: A Randomized Clinical Trial
Source: JAMA Psychiatry. 2025 Feb 12;82(4):406–14. doi: 10.1001/jamapsychiatry.2024.4801 (PMC11822603; doi:10.1001/jamapsychiatry.2024.4801)
Supplement: Supplement 1. — Trial Protocol [file jamapsychiatry-e244801-s001.pdf]

Study description and application to Regional Ethical Committee.

1. English title: Assessment and Treatment of Lung disease And Symptoms for people receiving Opioid Agonist Therapy (ATLAS4OAT)

2.1 Introduction

Patients with substance use disorders receiving opioid agonist treatment (OAT) are a group with a high disease burden, reduced quality of life, and a high risk of premature death [1, 2]. Lung disease and smoking constitute a significant part of the disease burden [3], with tobacco smoking alone contributing annually to around 7 million deaths and a loss of approximately 171 million disability-adjusted life years [3]. Chronic obstructive pulmonary disease (COPD) and respiratory infections, along with cardiovascular diseases, make up the majority of the disease burden related to tobacco smoking. The disease burden of COPD accounts for over 3 million deaths with a loss of around 50 million disability-adjusted life years [4]. In Norwegian population-based samples, the prevalence of COPD has been observed to be between 14 and 19 percent in the general population [5, 6]. The disease is characterized by shortness of breath and periods of exacerbations of respiratory symptoms. These periods worsen both lung function and quality of life and result in significant societal costs [7].

Among individuals receiving OAT treatment, more than ¾ smoke tobacco, and the prevalence of lung disease is significantly higher [8]. However, studies suggest that a large proportion of these individuals are interested in quitting smoking or reducing smoking [8], but the availability of these measures is often limited, and the measures are rarely well adapted to the setting. Several studies have tested various types of smoking cessation measures in recent years in different populations. These studies indicate that common smoking cessation measures are somewhat more challenging to succeed with among those with substance use disorders than among the general population [9, 10], but that such measures are still cost-effective in comparable settings in the UK [11]. Cannabis smoking constitutes a significantly smaller part of the global disease burden but is frequent among this group and also appears to contribute to some negative consequences for the lungs [12].

Although it seems more challenging to achieve total smoking cessation among those receiving OAT treatment [8], harm reduction is often achievable with known measures [8, 9, 13]. Nicotine replacement therapy (nicotine patches, nicotine gum, etc.), especially when combined with motivational interviewing, appears particularly promising in this group [13]. Recently, e-cigarettes in a normal population were compared to nicotine replacement therapy in a study that favored the use of e-cigarettes, including a reduction in cough and phlegm symptoms [14]. However, very few intervention studies with strong designs have been conducted among substance users.

Therefore, there is a need for better studies on how to succeed in reducing the somatic disease burden, such as lung disease, among those with opioid dependence receiving medication-assisted rehabilitation.

2.2 Benefits for Patient Treatment and Societal Value

Substance users have difficulty accessing essential health services, but targeted measures, such as treating chronic infections among substance users, can be effective [15]. To reach patients with substance use disorders, it has proven necessary to use methods characterized by more interdisciplinarity, accessibility, and closer follow-up than what is usually used in the healthcare system [16-18]. This is also supported by preliminary results from the INTRO-HCV study, which looked at the effect of integrating hepatitis C treatment into OAT treatment [19]. The results of this study suggest that integrating somatic health services into substance use treatment can improve health outcomes. The Helse Vest strategy (https://helse-vest.no/om-oss/mal-og-strategiar/helse2035) emphasizes that health services must be organized based on the patient to provide good treatment and that mental health care, somatic treatment, and substance use treatment should be closely integrated. This is also in line with the 2019 assignment documents from the Ministry of Health and Care Services to the regional health authorities for the care of somatic health and substance use treatment, providing clear guidelines for better integration in diagnosis, treatment, and follow-up with interdisciplinary collaboration.

Such an integrated treatment model for patients in OAT has been established in recent years at the Department of Addiction Medicine in Bergen and Stavanger with community-based OAT outpatient clinics where poorly functioning patients are followed up more or less daily by health and social care personnel linked to supervised intake of OAT medications [2]. This is a group of patients with a high disease burden who have been largely unable to benefit from other services in the healthcare system. Each OAT outpatient clinic is associated with a multidisciplinary team consisting of, among others, a chief physician and a physician specializing in addiction medicine, nurses, social workers, and psychologists. This provides a unique opportunity to conduct systematic, course-oriented, and clinically close research due to the clinical infrastructure with a contact surface of nearly ten thousand patient contacts per month linked to over a thousand individuals receiving OAT.

In recent years, we have had close collaboration with infectious and gastroenterological specialists targeting chronic infections in OAT. BAR (Bergen Addiction Research) now coordinates the INTRO-HCV study, where around 650 patients in Bergen and Stavanger have been mapped, and over 200 people have been treated for hepatitis C in a randomized controlled study (results will be analyzed this coming fall).

2.3 Regional Anchoring

We now think it may be necessary to further expand the focus with collaboration with pulmonary specialists to improve the management of somatic health in substance use treatment. BAR, together with KORFOR (Regional Competence Center for Substance Use Research in Helse Vest) and researchers with a pulmonary background, will establish a research initiative focusing on lung disease among substance users.

With both Helse Stavanger, Helse Bergen, BAR, and KORFOR strongly represented in the region, all relevant environments working with outpatient OAT treatment in Helse Vest will be represented. Our hypothesis is that a multimodal and integrated assessment and treatment model offered to substance users at OAT outpatient clinics will improve functional levels and quality of life. We aim to test this with a treatment study after mapping the prevalence of lung-related disease and symptoms among patients in OAT.

3. Research Questions and Goals

The overall goal is to build a research initiative and infrastructure research related to somatic disorders among substance users (ATLAS4LAR). The main goal through this infrastructure is to optimize the treatment of both somatic and mental health aspects among substance users. The sub-goals are divided into four work packages:

1. Calculate the incidence and prevalence of various lung diseases, including chronic obstructive pulmonary disease and pulmonary fibrosis, among people with substance use disorders with follow-up at OAT outpatient clinics in Bergen and Stavanger (work package 1, [WP1], see timeline below in figure 1)
2. Map risk factors for lung disease, including quantifying the effect of tobacco and cannabis among people with follow-up at OAT outpatient clinics in Bergen and Stavanger, as well as through registry analyses (work package 2, [WP2])
3. Test a multimodal intervention aimed at lung disease and risk factors for lung disease among people with follow-up at OAT outpatient clinics in Bergen and Stavanger (work package 3, [WP3])
4. Scale up and evaluate multimodal treatment through an intervention study with factorial design (work package 4, [WP4])

See the planned timeline for the work packages in figure 1 below.

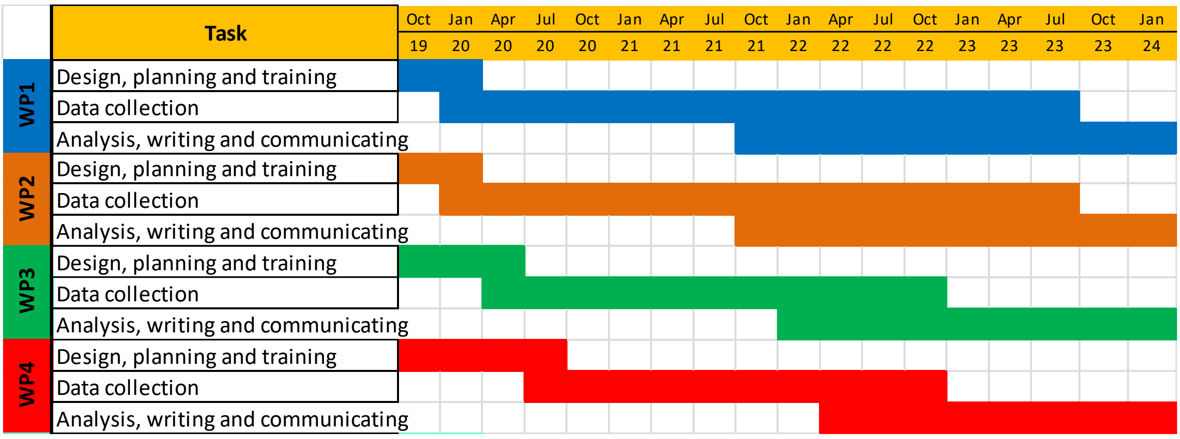

Figure 1: Timeline for the ATLAS4LAR Studies. WP: Work Package (see below for details on the work packages)

4. Implementation

The project will largely be integrated with and linked to clinical work, and much of the data collection infrastructure can be connected to the ongoing data collection for the OAT health registry and the INTRO-HCV study, where data collection will continue until mid-2020 [19]. The close clinical integration will make it feasible to carry out the research project if the application is approved.

4.1 Study Design, Method Selection, and Analyses

The ATLAS4LAR studies consist of four sub-studies/work packages, which are described in more detail below.

Work Package 1: Observational Study for Incidence and Prevalence of Lung Disease The first phase will be conducted as a prospective cohort without intervention. The goal of this work

package is to map the prevalence of chronic obstructive pulmonary disease (COPD), pulmonary fibrosis, other lung diseases, and lung-related symptoms among people with substance use disorders receiving follow-up at the OAT outpatient clinics in Bergen and Stavanger. We aim to map approximately 600-700 patients in OAT Bergen and around 200 patients in OAT Stavanger. The planned start for data collection is December 2019, and all patients at the selected clinics will be invited to participate in this survey. Semi-annual data collection will then be conducted, allowing for the assessment of changes in lung-related symptoms/complaints. Above is a timeline describing the planned timeline for the study (Figure 1), and below is the timeline of the surveys in the work packages (Figure 2).

In the target population, we plan to expand an already established annual survey with the collection of relevant clinical data (OAT health survey). The health surveys will include clinical examination, spirometry, blood tests, and the collection of self-reported information. The information obtained from the annual health survey for each participant who consents to research participation will be used for both clinical purposes and research analyses as described below. Patient follow-up will be conducted by a physician in specialization or a consultant in addiction medicine in collaboration with a nurse, psychologist, and social worker, and lung mapping will be done by/in collaboration with specialists in lung diseases. Data collection is streamlined by ensuring that the information used in the research project is clinically relevant and will be part of standard treatment follow-up at the OAT outpatient clinics. Since the information collection, clinical examination, and blood sampling will be part of the OAT treatment and simultaneously used for the research project, this will impose relatively little additional workload on the healthcare personnel and patients involved, although data registration will require some extra effort beyond normal outpatient clinic operations. The additional effort is planned to be carried out by hiring research nurses in Bergen and Stavanger, in addition to the Department of Addiction Medicine in Bergen also funding nursing positions equivalent to approximately two full-time equivalents per year.

Spirometry will be performed using the EasyOne Air spirometer, and at least one reversibility test with a bronchodilator (400 µg inhaled salbutamol) will be conducted. Chronic obstructive pulmonary disease is detected by spirometry when the ratio between forced expiratory volume in one second (FEV1) and forced vital capacity (FVC) is reduced to below 0.7 after the administration of inhaled bronchodilators. Spirometry will measure both forced expiratory volume in the first second (FEV1) and the ratio between FEV1 and total lung volume (FVC). We will also refer for chest X-rays and, if clinically indicated, computed tomography (CT) scans of the lungs. Extended blood tests will be taken as part of a clinical annual check-up and include the mapping of chronic infections such as hepatitis and HIV, as well as biochemical tests such as hematology, thyroid function tests, glucose, glycated hemoglobin (HbA1c), and transaminases. Research-trained nurses will take blood samples, which will be sent for analysis at the Laboratory for Clinical Biochemistry at Haukeland University Hospital, and will also conduct liver examinations with elastography (FibroScan®) and electrocardiography of the heart.

In addition to blood tests, a clinical examination will be conducted to map other somatic diseases, including measurements of blood pressure and weight. A clinical interview will also be conducted, including questions from standardized questionnaires mapping lung-related complaints (Medical Research Council dyspnea scale, MRC, and Chronic Obstructive Lung Disease Assessment Test, CAT), the extent to which patients use various substances (modified from DUDIT-E), quality of life (EQ-5D-5L), fatigue (FAS), psychological complaints (HSCL-10/25), physical performance/fitness, infection-related risk behavior, and dietary patterns. Information collected relevant to both clinical treatment and the research project will be stored in parallel in the electronic patient journal and a

research database using the CheckWare tool. Helse Vest already has a license for CheckWare, which offers integration into the electronic patient journal.

Work Package 2: Risk Factors for Lung Disease Among Substance Users The goal of work package 2 is to examine risk factors for lung disease among people with substance use disorders. We will do this by examining changes in various exposures and how this affects lung function and respiratory symptoms over time using, among other things, mixed models regression analyses. Register-based analyses are also planned using, among others, the Norwegian Patient Registry (NPR), the Prescription Registry (NorPD), and the Cause of Death Registry to examine lung comorbidity among people with substance use disorders. We will look at diagnosed lung disease among those receiving OAT treatment, the use of other medications including inhalation medications, the frequency of hospitalizations, and deaths.

	Task	Recruitment	3 months	6 months	9 months	12 months	18 months	24 months	36 months
WP1 & 2	Initial interview	x							
	Follow-up interviews			x		x		x	x
	Blood sample collection and	x				x		x	x
	Clinical examination	x		x		x	x	x	x
WP3&4	Initial interview	x							
	Follow-up interviews		x	x	x	x	x	x	x
	Blood sample collection and	x		x		x		x	x
	Clinical examination	x	x	x	x	x	x	x	x

Figure 2: Data Collection Plan for Work Package 1+2 (WP1&2) and 3+4 (WP3&4).

Work Package 3: Testing/Piloting a Multimodal Intervention In Work Package 3, we will test the effect of various interventions focusing on improving lung function and reducing respiratory symptoms. The measures we want to test will particularly address the following aspects:

- Smoking cessation/reduction/harm reduction for tobacco smoking
- Improving physical function through activity/exercise guidance and dietary measures (BAReAktiv)
- Fruit smoothie (FruktBAR)

We have piloted three intervention pilots:

1. Integration of substitution therapy with nicotine patches/gum
2. Activity intervention with activity groups meeting 3 times per week (BAReAktiv)
3. Dietary intervention with fruit smoothies (FruktBAR). This will also include validation of a dietary survey using an online form.

The intervention pilots were conducted over 8 weeks and evaluated using mixed methods, combining structured questionnaires and qualitative interviews (focus groups and individual) with both participants and involved healthcare personnel. Audio recordings of these interviews will be transcribed and analyzed qualitatively. Approximately 20-25 people are planned to be included in each of the intervention pilots.

A team consisting of a nurse and a doctor will assess and guide the measures and choice of interventions, which will be evaluated with the following main outcomes:

- Change in tobacco and cannabis use (self-reported)

- Change in FEV1 and FEV1/FVC ratio (spirometry assessed)
- Improvement in self-reported physical performance (self-reported and tested)

Inclusion Criteria: Individuals receiving OAT treatment who smoke tobacco daily and/or cannabis, and consent to participate in harm reduction intervention.

Work Package 4: Randomized Clinical Trial Following this, we aim to scale up to a factorial-design randomized clinical trial to evaluate the effects of the various interventions (see above in Work Package 3). We will include 300 people in the intervention study, where 3/8 will be offered one of the interventions, 3/8 will be offered two interventions, 1/8 will receive standard treatment without additional interventions, and 1/8 will be offered all interventions.

In summary, the interventions will be as follows:

1. Activity intervention with activity groups meeting twice a week for 16 weeks (BAReAktiv)
2. Provision of an integrated smoking cessation package with motivational interviews and nicotine replacement products such as nicotine patches and gum over a 12-week period, which can be extended up to 12 weeks with successful smoking cessation or significant reduction in smoking
3. Dietary intervention with fruit smoothies (FruktBAR) with the distribution of fruit smoothie products (250 ml daily with a ready-made product) over 16 weeks, distributed weekly.

The effect of the interventions will be evaluated in week 16 (12-20) using a combination of questionnaires with survey forms and blood tests mentioned in Work Package 1. Inclusion criteria will be that the person receives OAT treatment, wishes to participate in each of the relevant interventions, and consents to this. For nicotine replacement products, it is also a criterion that they smoke tobacco daily, and for fruit smoothies, that they eat 3 or fewer servings of fruits/vegetables per day. Individuals allergic to nicotine replacement products and fruit smoothies are excluded from these interventions, as are those with poorly regulated diabetes. The details of the interventions are otherwise described in clinicaltrials.org (NCT05242848, 155386/REK-B, NCT05229770). We plan to use similar outcome measures in Work Package 4 as in Work Package 3.

4.2 Organization and Collaboration

The project will be carried out in close collaboration between Helse Bergen and Helse Stavanger under the auspices of Bergen Addiction Research (BAR) at the Department of Addiction Medicine, Haukeland University Hospital. Lars T. Fadnes is the project leader, and department director Else-Marie Løberg is the project manager. The project group is interdisciplinary and includes many with broad clinical experience in addiction medicine and pulmonary research. The project group consists of:

- **Lars T. Fadnes (MD, PhD)** is a specialist in general medicine and senior researcher at the Department of Addiction Medicine in Helse-Bergen, associate professor at the University of Bergen, and leader of the Bergen Addiction Research Group (BAR). He has research experience in addiction-related research, hepatitis C, HIV, and nutrition, and has worked with controlled clinical studies, cohort studies, and registry data. Fadnes will lead and coordinate the study and supervise PhD candidates.

- **Torgeir Gilje Lid (MD, PhD)** is a specialist in general medicine and works as a senior physician/research leader (50%) at the Regional Competence Center for Substance Use Research in Helse Vest (KORFOR) in Helse-Stavanger, postdoctoral fellow (50%) at Helse Vest, and associate professor (20%) at the University of Stavanger. He has research experience in addiction-related research in general medicine and hospitals and will be involved in the design, supervision, implementation, evaluation, and writing of the study.
- **Tesfaye Madebo (MD, PhD)** is a senior physician at the Lung Section at Stavanger University Hospital. He has researched tuberculosis and lung function testing and will be involved in the design, implementation, and evaluation of the study and supervise PhD candidates.
- **Hege Tønnesen (MD)** is a senior physician at the Department of Addiction Medicine and is part of the addiction medicine forum focusing on lung disease among substance users. She will be involved in the design, implementation, and evaluation of the study.
- **Tomas Mikal Lind Eagan (MD, PhD)** and **Rune Nielsen (MD, PhD)** are senior physicians at the Lung Department, Haukeland University Hospital, and professors/associate professors at the University of Bergen. They have research experience in pulmonary research, including chronic obstructive pulmonary disease, and have worked with cohort studies, microbiome, and genetics research. Eagan and Nielsen will contribute to the design, analysis, and writing of the work.
- **Nancy Laura Ortega Maldonado (MD)** is a senior physician at the Department of Addiction and Dependency Treatment in Stavanger and has contributed to the implementation of the INTRO-HCV project. She will be involved in the design, implementation, and evaluation of the study.
- **Rune Blomhoff (professor)** and **Hege Berg Henriksen (PhD candidate)** at the University of Oslo both work with nutrition and will contribute to the design, analysis, and writing of the fruit smoothie intervention and validation of dietary assessment in the study.
- **Kjell Arne Johansson (MD, PhD)** is a senior physician and senior researcher at the Department of Addiction Medicine in Helse-Bergen and professor at the University of Bergen. He works clinically with addiction medicine and health priorities. He has particular expertise in evaluating the introduction of new technology in healthcare, has a large international research network, and has worked with several epidemiological methods. He will be involved in the design and analysis of the study.
- **Christer Frode Aas (MD)**, **Jørn Henrik Vold (MD)**, and **Fatemeh Chalabianloo (MD)** are doctors and PhD candidates at the Department of Addiction Medicine in Helse-Bergen and work with addiction medicine with clinical experience from OAT and will contribute to the implementation and analysis of the study. Similarly, **Einar Furulund** and **Karl Trygve Druckrey-Fiskaaen** are PhD candidates involved in this project.
- **Else-Marie Løberg (MD, PhD)** is the department director at the Department of Addiction Medicine in Helse-Bergen and professor at the University of Bergen. She works with addiction medicine, neurocognition, psychosis, and user involvement and leads/has led research projects related to brain function, psychosis, trauma, addiction, genetics, treatment studies, and patient-centered research. Løberg will be involved in evaluating mental health outcomes in the study and is the project manager.
- **Vibeke Bråthen Buljovcic, Jan Tore Daltveit, Maria Kålås Olsvold, Mette Hegland Nordbotn, Per Gundersen, and Ewa Joanna Wilk** are research nurses with solid experience in both clinical follow-up and research follow-up. The first mentioned is the coordinator for the study, and in addition, **Tone Lise Eielsen** is the head of the OAT outpatient clinic where the study will take place in Stavanger. All will be central in the design, interpretation, and implementation of the study.

- **Marianne Pierron, Christine Sundal, Eivin Dahl, and Ole Jørgen Lygren** are user representatives with important experience-based competence. They will be central in the design, interpretation, and dissemination of the study.
- **Siv-Elin Leirvåg Carlsen** and **Silvia Eiken Alpers** are PhD candidates at the Department of Addiction Medicine with expertise related to substance use disorders. Both will be involved in the design, interpretation, and dissemination of the study.
- We will also recruit an international advisory board that will be involved in the planning and evaluation of the studies and will have at least three meetings during the project period, including online and telephone meetings. At least one of the meetings will be organized as a combined advisory meeting for the project group and partners in connection with a national or international seminar/conference on substance use disorders and somatic health.
-

4.3 Budget

- Much of the work will be closely linked to clinical work, but the effort will require some additional work related to data collection, where we wish to hire research nurses in Bergen and Stavanger (one in Stavanger and one in Bergen, as well as one for coordination). We are also applying for a PhD candidate in Bergen and one in Stavanger associated with the initiative, as well as a smaller sum for some buyout of a post-doc researcher in a 20% position. We will have two user representatives who will be employed 20% during the project period. In addition, there will be expenses for organizing collaboration meetings, publication fees for open access publishing, linking to registry data, CheckWare adaptation, and the purchase of EasyOne Air spirometers. There are also some costs for the purchase of nicotine substitution (with nicotine gum, smoking patches, etc.). Most of the project group members are employed by health trusts and/or universities and will have salary funds from there during the work with implementation, data analysis, and writing of articles. Overall, we will need NOK 18,974,000 for the research project over the project period (see details in the web application). The project is funded by strategic research funds from Helse Vest.
-

4.4 Plan for Progress and Publication

- Figure 1 shows the timeline for the ATLAS4LAR studies, including the schedule and milestones for the project's implementation. Since we have a study with ongoing data collection until spring 2020, we plan to start data collection for Work Package 1 shortly after the application is approved. We plan to complete the writing and analysis for the study by the end of 2024. The activity plan is also attached to the application form (eSøknad).
- **Dissemination of Results and Publication** The results of the various projects will be published as approximately 15 articles in peer-reviewed journals in accordance with ICMJE guidelines [20]. The project and findings will be presented at relevant international and national conferences. Several members of the research group have extensive experience in communicating research results to the public through both mass media, social media, information targeted at patient groups, and teaching for students, doctors, and other healthcare personnel.
- **Communication to Users** Users, both patients and healthcare personnel, will be included in all phases from planning to implementation and dissemination of our research results. The results will be communicated to the participants. In addition, we also plan our own event in collaboration with users with targeted professional dissemination combined with a concert.

- ## 4.5 Plan for Implementation
- If the interventions prove to be effective, we plan to work to scale up these interventions nationally through dissemination of results, contact with health authorities and health trusts, work with guidelines, and contributions to training and education. We assume that the research will also be highly relevant for other health trusts in their work with substance users. The project is considered to have significant international relevance and innovation potential.
-
- ## 5. User Involvement
- We will collaborate with the user organization ProLAR Nett (http://prolar.no), including representative and co-researcher Ole Jørgen Lygren, who has a strong focus on the importance of scaling up treatment offers for OAT patients. We will also collaborate with A-larm. KORFOR has established good cooperation with A-larm in both research and professional development, and A-larm (including Jan Ivar Ekberg) will contribute to the planning and implementation of the studies. Two co-researchers in 20% positions with user backgrounds and user representatives will also be involved in the development of surveys/questionnaires and interventions, interpretation, and writing of results.
-
- ## 6. Ethical Considerations
- The study is not expected to have negative consequences for the participants, but if adverse events occur, we will tailor necessary treatment in close collaboration with clinicians who follow up the patient at the OAT outpatient clinic or relevant hospital departments. The study will be conducted in accordance with the Helsinki Declaration and will be submitted for approval by the Regional Ethical Committee West. We have already received ethical approval for the biobank and a OAT health registry (REK 2016/1080). Informed consent will be obtained in writing from participants after they have received both oral and written information about the study. Data will be stored securely and in encrypted format, and the data files used will be de-identified/anonymized. For registry linkages, permission will be sought after dialogue with the data protection officer and registry owners (and a Data Protection Impact Assessment will be conducted). The intervention study will also be registered on https://clinicaltrials.gov.
-
- ## 7. References

1. Forouzanfar MH, Alexander L, Anderson HR, et al. Global, regional, and national comparative risk assessment of 79 behavioural, environmental and occupational, and metabolic risks or clusters of risks in 188 countries, 1990-2013: a systematic analysis for the Global Burden of Disease Study 2013. *Lancet*. Dec 5 2015;386(10010):2287-323. doi:10.1016/s0140-6736(15)00128-2
2. Collaborators GBDCoD. Global, regional, and national age-sex-specific mortality for 282 causes of death in 195 countries and territories, 1980-2017: a systematic analysis for the Global Burden of Disease Study 2017. *Lancet*. Nov 10 2018;392(10159):1736-1788. doi:10.1016/S0140-6736(18)32203-7
3. Inoue-Choi M, Christensen CH, Rostron BL, et al. Dose-Response Association of Low-Intensity and Nondaily Smoking With Mortality in the United States. *JAMA Netw Open*. Jun 1 2020;3(6):e206436. doi:10.1001/jamanetworkopen.2020.6436
4. Inoue-Choi M, Hartge P, Park Y, Abnet CC, Freedman ND. Association Between Reductions of Number of Cigarettes Smoked per Day and Mortality Among Older Adults

in the United States. *Am J Epidemiol*. Feb 1 2019;188(2):363-371. doi:10.1093/aje/kwy227
5. Hayes RD, Chang CK, Fernandes A, et al. Associations between substance use disorder sub-groups, life expectancy and all-cause mortality in a large British specialist mental healthcare service. *Drug Alcohol Depend*. Oct 1 2011;118(1):56-61. doi:10.1016/j.drugalcdep.2011.02.021
6. Lewer D, Jones NR, Hickman M, Nielsen S, Degenhardt L. Life expectancy of people who are dependent on opioids: A cohort study in New South Wales, Australia. *J Psychiatr Res*. Nov 2020;130:435-440. doi:10.1016/j.jpsychires.2020.08.013
7. Rehm J, Manthey J, Shield KD, Ferreira-Borges C. Trends in substance use and in the attributable burden of disease and mortality in the WHO European Region, 2010-16. *Eur J Public Health*. Aug 1 2019;29(4):723-728. doi:10.1093/eurpub/ckz064
8. Westman J, Wahlbeck K, Laursen TM, et al. Mortality and life expectancy of people with alcohol use disorder in Denmark, Finland and Sweden. *Acta Psychiatr Scand*. Apr 2015;131(4):297-306. doi:10.1111/acps.12330
9. Aas CF, Vold JH, Skurtveit S, et al. Health-related quality of life of long-term patients receiving opioid agonist therapy: a nested prospective cohort study in Norway. *Subst Abuse Treat Prev Policy*. Sep 3 2020;15(1):68. doi:10.1186/s13011-020-00309-y
10. Guydish J, Passalacqua E, Pagano A, et al. An international systematic review of smoking prevalence in addiction treatment. *Addiction*. Feb 2016;111(2):220-30. doi:10.1111/add.13099
11. Bech AB, Clausen T, Waal H, Delaveris GJM, Skeie I. Organ pathologies detected post-mortem in patients receiving opioid agonist treatment for opioid use disorder: a nation-wide 2-year cross-sectional study. *Addiction*. Apr 2022;117(4):977-985. doi:10.1111/add.15705
12. Mehta S, Parmar N, Kelleher M, et al. COPD and asthma in patients with opioid dependency: a cross-sectional study in primary care. *NPJ Prim Care Respir Med*. Jan 14 2020;30(1):4. doi:10.1038/s41533-019-0161-7
13. Apollonio D, Philipps R, Bero L. Interventions for tobacco use cessation in people in treatment for or recovery from substance use disorders. *Cochrane Database Syst Rev*. Nov 23 2016;11:CD010274. doi:10.1002/14651858.CD010274.pub2
14. Guydish J, Kapiteni K, Le T, Campbell B, Pinsker E, Delucchi K. Tobacco use and tobacco services in California substance use treatment programs. *Drug Alcohol Depend*. Sep 1 2020;214:108173. doi:10.1016/j.drugalcdep.2020.108173
15. Vlad C, Arnsten JH, Nahvi S. Achieving Smoking Cessation Among Persons with Opioid Use Disorder. Review. *CNS Drugs*. 01 Apr 2020;34(4):367-387.
16. Yee A, Hoong MC, Joyce YC, Loh HS. Smoking Cessation Among Methadone-Maintained Patients: A Meta-Analysis. *Subst Use Misuse*. Jan 28 2018;53(2):276-285. doi:10.1080/10826084.2017.1342661
17. Notley C, Gentry S, Livingstone-Banks J, Bauld L, Perera R, Hartmann-Boyce J. Incentives for smoking cessation. *Cochrane Database Syst Rev*. Jul 17 2019;7(7):Cd004307. doi:10.1002/14651858.CD004307.pub6
18. Lindson-Hawley N, Thompson TP, Begh R. Motivational interviewing for smoking cessation. *Cochrane Database Syst Rev*. Mar 2 2015;(3):Cd006936. doi:10.1002/14651858.CD006936.pub3
19. Patnode CD, Henderson JT, Coppola EL, Melnikow J, Durbin S, Thomas RG. Interventions for Tobacco Cessation in Adults, Including Pregnant Persons: Updated Evidence Report and Systematic Review for the US Preventive Services Task Force. *Jama*. Jan 19 2021;325(3):280-298. doi:10.1001/jama.2020.23541

20. Park ER, Perez GK, Regan S, et al. Effect of Sustained Smoking Cessation Counseling and Provision of Medication vs Shorter-term Counseling and Medication Advice on Smoking Abstinence in Patients Recently Diagnosed With Cancer: A Randomized Clinical Trial. *Jama*. Oct 13 2020;324(14):1406-1418. doi:10.1001/jama.2020.14581
21. Hall SM, Humfleet GL, Gasper JJ, Delucchi KL, Hersh DF, Guydish JR. Cigarette Smoking Cessation Intervention for Buprenorphine Treatment Patients. *Nicotine Tob Res*. Apr 2 2018;20(5):628-635. doi:10.1093/ntr/ntx113
22. Strand BH, Dalgard OS, Tambs K, Rognerud M. Measuring the mental health status of the Norwegian population: a comparison of the instruments SCL-25, SCL-10, SCL-5 and MHI-5 (SF-36). *Nord J Psychiatry*. 2003;57(2):113-8. doi:10.1080/08039480310000932
23. Vieira EB, Degani-Costa LH, Amorim BC, et al. Modified BODE Index to Predict Mortality in Individuals With COPD: The Role of 4-Min Step Test. *Respir Care*. Jul 2020;65(7):977-983. doi:10.4187/respcare.06991
24. Peak J, Goranitis I, Day E, Copello A, Freemantle N, Frew E. Predicting health-related quality of life (EQ-5D-5 L) and capability wellbeing (ICECAP-A) in the context of opiate dependence using routine clinical outcome measures: CORE-OM, LDQ and TOP. *Health Qual Life Outcomes*. May 30 2018;16(1):106. doi:10.1186/s12955-018-0926-7
25. Vold JH, Gjestad R, Aas CF, Meland E, Johansson KA, Fadnes LT. Validation of a three-item Fatigue Severity Scale for patients with substance use disorder: a cohort study from Norway for the period 2016-2020. *Health Qual Life Outcomes*. Mar 2 2021;19(1):69. doi:10.1186/s12955-021-01708-w
26. Hajiro T, Nishimura K, Tsukino M, Ikeda A, Koyama H, Izumi T. Analysis of clinical methods used to evaluate dyspnea in patients with chronic obstructive pulmonary disease. *Am J Respir Crit Care Med*. Oct 1998;158(4):1185-9. doi:10.1164/ajrccm.158.4.9802091
27. Craig CL, Marshall AL, Sjöström M, et al. International physical activity questionnaire: 12-country reliability and validity. *Med Sci Sports Exerc*. Aug 2003;35(8):1381-95. doi:10.1249/01.Mss.0000078924.61453.Fb
28. Calvert M, Kyte D, Mercieca-Bebber R, et al. Guidelines for Inclusion of Patient-Reported Outcomes in Clinical Trial Protocols: The SPIRIT-PRO Extension. *Jama*. Feb 6 2018;319(5):483-494. doi:10.1001/jama.2017.21903
29. Welch VA, Norheim OF, Jull J, Cookson R, Sommerfelt H, Tugwell P. CONSORT-Equity 2017 extension and elaboration for better reporting of health equity in randomised trials. *Bmj*. Nov 23 2017;359:j5085. doi:10.1136/bmj.j5085
30. Moher D, Schulz KF, Altman DG. The CONSORT statement: revised recommendations for improving the quality of reports of parallel-group randomised trials. *Lancet*. Apr 14 2001;357(9263):1191-4.
31. Hartmann-Boyce J, Chepkin SC, Ye W, Bullen C, Lancaster T. Nicotine replacement therapy versus control for smoking cessation. *Cochrane Database Syst Rev*. May 31 2018;5(5):Cd000146. doi:10.1002/14651858.CD000146.pub5
32. Nahvi S, Adams TR, Ning Y, Zhang C, Arnsten JH. Effect of varenicline directly observed therapy versus varenicline self-administered therapy on varenicline adherence and smoking cessation in methadone-maintained smokers: a randomized controlled trial. *Addiction*. Apr 2021;116(4):902-913. doi:10.1111/add.15240
33. Fadnes LT, Aas CF, Vold JH, et al. Integrated treatment of hepatitis C virus infection among people who inject drugs: A multicenter randomized controlled trial (INTRO-HCV). *PLoS Med*. Jun 2021;18(6):e1003653. doi:10.1371/journal.pmed.1003653

34. Vijayaraghavan M, Elser H, Frazer K, Lindson N, Apollonio D. Interventions to reduce tobacco use in people experiencing homelessness. *Cochrane Database Syst Rev*. Dec 3 2020;12(12):Cd013413. doi:10.1002/14651858.CD013413.pub2
35. Guideline for good clinical practice E6(R2) (2018).
36. OECD Principles on Good Laboratory Practice (1998).
37. Gamble C, Krishan A, Stocken D, et al. Guidelines for the Content of Statistical Analysis Plans in Clinical Trials. *Jama*. Dec 19 2017;318(23):2337-2343. doi:10.1001/jama.2017.18556
38. Morris CD, Garver-Apgar CE. Nicotine and Opioids: a Call for Co-treatment as the Standard of Care. *J Behav Health Serv Res*. Oct 2020;47(4):601-613. doi:10.1007/s11414-020-09712-6
39. Druckrey-Fiskaaen KT, Furulund E, Daltveit JT, et al. Integration of smoking cessation into standard treatment for patients receiving opioid agonist therapy who are smoking tobacco: protocol for a randomised controlled trial (ATLAS4LAR). *Trials*. Aug 17 2022;23(1):663. doi:10.1186/s13063-022-06560-x
40. Sedgwick P. Spearman's rank correlation coefficient. *Bmj*. Nov 28 2014;349:g7327. doi:10.1136/bmj.g7327

-

Decision Letter - Change Approved by Regional Ethical Committee (REK)

Region: REK sør-øst C
Case Handler: Anders Strand
Phone: 22 84 55 11
Date: 05.04.2022
Reference Number: 155386

Project Application: Mapping and Treatment of Lung Disease in Medication-Assisted Rehabilitation (ATLAS4LAR)
Applicant: Lars Thore Fadnes
Responsible Institution: Helse Bergen HF - Haukeland University Hospital

Applicant's Description:
Substance abusers have difficulty accessing essential health services, but targeted measures, such as treating chronic infections in substance abusers, can be effective. To reach patients with substance use disorders, it has proven necessary to use methods characterized by more interdisciplinarity, accessibility, and closer follow-up than what is usually used in the healthcare system. This is supported by preliminary results from the INTRO-HCV study, which looked at the effect of integrating hepatitis C treatment into OAT treatment. The results suggest that integrating somatic health services into substance abuse treatment can improve health outcomes. The Helse Vest strategy emphasizes that health services must be organized based on the patient to provide good treatment and that mental health care, somatic treatment, and substance abuse treatment should be closely integrated. Such an integrated treatment model for patients in OAT has been established in recent years at the Department of Addiction Medicine in Bergen and Stavanger with community-based OAT clinics where poorly functioning patients are followed up more or less daily by health and social professionals. This is a group of patients with a high disease burden who have been unable to benefit from other services in the healthcare system. Each OAT clinic has a multidisciplinary team consisting of, among others, a senior physician and a specialist in addiction medicine, nurses, social workers, and psychologists. This provides a unique opportunity to conduct systematic, process-oriented, and clinically relevant research due to the clinical infrastructure with a contact surface of nearly ten thousand patient contacts per month linked to over a thousand individuals receiving OAT. In recent years, we have had close cooperation with infection and gastroenterology specialists targeting chronic infections in OAT. BAR (Bergen Addiction Research) now coordinates the INTRO-HCV study, where around 650 patients in Bergen and Stavanger have been mapped, and over 200 people have been treated for hepatitis C in a randomized controlled study. We are now expanding the focus to heart and lung disease to improve the management of somatic health in substance abuse treatment. Our hypothesis is that a multimodal and integrated assessment and treatment model offered to substance abusers at OAT clinics will improve functional levels and quality of life. We want to test this with a treatment study after mapping the prevalence of lung-related diseases and symptoms among patients in OAT.

Issues and Goals:
The overall goal is to build a research initiative and infrastructure for process research related to somatic disorders among substance abusers (ATLAS4LAR). The main goal is to optimize the treatment of both somatic and mental health aspects among substance abusers through this infrastructure. The sub-goals are divided into four work packages:

1. Calculate the incidence and prevalence of various lung diseases, including chronic obstructive pulmonary disease and lung fibrosis, among people with substance abuse with follow-up at OAT clinics in Bergen and Stavanger (work package 1).
2. Map risk factors for lung disease, including quantifying the effect of tobacco and cannabis among people with follow-up at OAT clinics in Bergen and Stavanger and through registry analyses (work package 2).
3. Test a multimodal intervention targeting lung disease and risk factors for lung disease among people with follow-up at OAT clinics in Bergen and Stavanger, evaluated qualitatively (work package 3).
4. Scale up and evaluate multimodal treatment through an intervention study with factorial design (work package 4).

REK's Assessment:
The requested changes include:

- Einar Furulund (Helse Stavanger HF) is included as a project collaborator. The committee has no ethical objections to this.
- The protocol is updated with planned scaled-up versions of three interventions: an activity intervention, a smoking cessation program, and a diet-focused intervention. These are described on pages 5-7 in the attached updated protocol. The scaling up is based on positive experiences from completed pilots and appears well-founded, potentially useful, and justifiable to the committee. The committee therefore approves the requested changes.

Decision:
The committee has reviewed the change request and approves the requested changes under the authority of the Health Research Act § 11. The permission is granted on the condition that the project change is carried out as described in the project change notification and change protocol, and the provisions of the Health Research Act with regulations are followed. Please quote our reference number in correspondence.

Final Report:
The project leader must send a final report to REK on a separate form via the REK portal no later than 6 months after the end date 31.12.2029, cf. Health Research Act § 12. If the project does not start or is not carried out, this must also be reported via the final report form.

Application for Change:
If you wish to make significant changes in purpose, method, timeline, or organization, the project leader must send an application for change via the portal on a separate form to REK, cf. Health Research Act § 11.

Right to Appeal:
You can appeal REK's decision, cf. Public Administration Act § 28 ff. The appeal is sent on a separate form via the REK portal. The appeal deadline is three weeks from receiving this letter. If REK upholds the decision, REK will forward the appeal to the National Research Ethics Committee for Medicine and Health Sciences (NEM) for final assessment, cf. Research Ethics Act § 10 and Health Research Act § 10.

Best regards,
Jacob Hølen, Secretariat Leader, REK sør-øst
Anders Strand, Senior Advisor, REK sør-øst C

Copy to:
Helse Bergen HF - Haukeland University Hospital

Integration of Smoking Cessation Into Standard Treatment for Patients Receiving Opioid Agonist Therapy Who Are Smoking Tobacco: Protocol for a Randomised Controlled Trial (ATLAS4LAR)

Names protocol contributors

Karl Trygve Druckrey-Fiskaaen [1,2], Einar Furulund [1,2,3], Jan Tore Daltveit [1] Jørn Henrik Vold [1,2,4], Torgeir Gilje Lid [3], Tesfaye Madebo [1,5], Lars T. Fadnes [1,2] for the ATLAS4LAR Study Group[6]

Corresponding author, Karl Trygve Druckrey-Fiskaaen: karl.fiskaaen@uib.no

Abstract

Background: About 85% of patients receiving opioid agonist therapy (OAT) for opioid dependence are smoking tobacco. Although smoke-related pulmonary diseases are significant contributors to morbidity and mortality, few smoking cessation interventions are evaluated within this group, and few OAT patients are offered smoking cessation as an integrated part of their addiction treatment. This study protocol describes an integrated smoking cessation intervention aimed at patients receiving OAT and smoking tobacco.

Methods: This is a multicentre, randomised controlled clinical trial that will recruit 266 daily tobacco smoking patients receiving OAT in OAT outpatient clinics in Bergen and Stavanger, Norway. The patients randomised for the intervention arm will be offered smoking cessation therapy consisting of weekly brief behavioural interventions and prescription-free nicotine replacement products. In the control arm, patients will receive standard care without any added interventions related to smoking cessation.

The smoking cessation intervention includes psychoeducational techniques with components from motivational interviewing, and nicotine replacement products such as nicotine lozenges, patches and chewing gum. The duration of the intervention is 16 weeks, with the option of extending it with a further eight weeks. The main outcomes are measured at 16 weeks after initiation of the intervention, and sustained effects are evaluated one year after intervention initiation.

The primary outcome is smoking cessation verified by carbon monoxide (CO)-levels or at least a 50% reduction in the number of cigarettes smoked. Secondary outcomes are changes in psychological well-being, biochemical inflammation markers, changes in physical health, quality of life and fatigue.

Discussion: Integration of other treatments to standard OAT care improves adherence and completion rates providing another rationale for integrated smoking cessation treatment. Thus, if integrated smoking cessation treatment is superior to standard care, this trial provides important information on further scale-up.

Trial registration: ClinicalTrials.gov NCT05290025, Date of registry 22. March 2022

Key Words

Smoking cessation, nicotine replacement, opiate substitution treatment, behavioural intervention, substance abuse treatment centres.

Administrative information Note: the numbers in curly brackets in this protocol refer to SPIRIT checklist item numbers. The order of the items has been modified to group similar items (see http://www.equator-network.org/reporting-guidelines/spirit-2013-statement-defining-standard-protocol-items-for-clinical-trials/).

Title {1}	Integration of Smoking Cessation Into Standard Treatment for Patients Receiving Opioid Agonist Therapy Who Are Smoking Tobacco: Protocol for a Randomised Controlled Trial (ATLAS4LAR)
Trial registration {2a and 2b}.	Registered in ClinicalTrials.gov NCT05290025, Date of registry 22. March 2022
Protocol version {3}	Version 2, 14.07.2022<br>Corrections made to SPIRIT ITEMS: 5c, 8, 26b, 16a, 33, 20c, 25 and 32. In addition typographical errors have been corrected.
Funding {4}	The study was funded by Western Norway Regional Health Authority («Strategiske forskningsmidler» through ATLAS4LAR-project) with Department of Addiction Medicine, Haukeland University Hospital as responsible institution.
Author details {5a}	[1] Bergen Addiction Research, Department of Addiction Medicine, Haukeland University Hospital, Bergen, Norway

	[2] Department of Global Public Health and Primary Care, University of Bergen, Norway<br>[3] Centre for Alcohol and Drug Research, Stavanger University Hospital, Stavanger, Norway<br>[4] Division of Psychiatry, Haukeland University Hospital, Bergen, Norway.<br>[5] Department of Respiratory Medicine, Stavanger University Hospital, Stavanger, Norway<br>[6] List of Members of the ATLAS4LAR Study Group (see acknowledgement)
Name and contact information for the trial sponsor {5b}	Department of Addiction Medicine, Haukeland University Hospital, director Christian Ohldieck, Post box 1400, 5021 Bergen. Christian.ohldieck@helse-bergen.no
Role of sponsor {5c}	The sponsor had no role in study design, and will have no role in data collection and analysis, decision to publish, or preparation of the manuscript. The sponsor does and will not have ultimate authority over any of these activities.

Introduction

Background and rationale {6a}

Lung disease and tobacco smoking contribute to a high burden of disease [1]. Tobacco smoking alone leads to seven million deaths yearly and loss of 171 million disability-adjusted life-years worldwide [1]. Chronic obstructive lung disease (COPD), airway infections, and cardiovascular disease are highly linked to tobacco smoking and are the most important reasons for the global tobacco-related disease burden. Indeed, COPD alone causes three million deaths and loss of approximately 50 million

disability-adjusted life-years yearly [2]. Although smoking cessation provides the largest mortality reduction, reducing smoking intensity also diminishes mortality [3,4], providing a more feasible approach for some populations. Persons with Opioid Dependence syndrome experience higher morbidity, lower quality of life, and have a substantially shorter expected lifespan than the general population [5-9]. Among patients with opioid dependence receiving opioid agonist therapy (OAT), nearly 85% smoke tobacco [10]. The prevalence of lung diseases is as high as 63% in autopsy samples of OAT patients [11], whereas 21 % of patients on methadone as OAT medication in a primary care setting had diagnosis of COPD, asthma or both [12]. Thus, ceasing tobacco smoking is likely to provide a significant decrease in morbidity and mortality among opioid dependent patients.

A systematic review found varying effects of providing tobacco cessation therapy parallel to the treatment of dependence syndromes [13]. Although most patients with opioid dependence smoke tobacco, few are offered smoking cessation treatment [14]. Among patients with opioid use disorder who were offered smoking cessation interventions, previous studies have generally not succeeded in smoking cessation [15].
On the other hand, combined counselling and nicotine replacement therapy (NRT) may, to some extent, improve the smoking cessation rate [13]. For patients receiving methadone as OAT medication, combined behavioural therapy and NRT have provided higher smoking cessation rates [16]. Financial incentives and NRT show promising results among patients with opioid use disorders [15].

In the general population, incentives boost cessation rates [17]. Motivational interviewing, including sessions up to 20 minutes, seem to aid in smoking cessation [18]. Behavioural interventions alone or in combination with pharmacotherapy increase quit rates [19]. The combination of behavioural intervention and provision of cessation medication increased quit rates in newly diagnosed cancer patients [20]. Among OAT patients on buprenorphine combination treatment for smoking cessation increased quit attempts and motivation to stop smoking, as well [21].

No trial has yet tested the effects of a combined smoking cessation intervention integrated in the OAT on the smoking pattern, pulmonary health, physical fitness, and mental health. A recently conducted pilot study with a similar intervention indicates promising results (submitted). We will thus conduct

a multicentre randomised controlled trial to investigate the effect of combined smoking cessation intervention administered weekly for up to 24 weeks on smoking patterns, psychological well-being, and physical tests.

Objectives {7}

This paper presents the protocol of the ATLAS4LAR smoking cessation intervention. The primary objective is to assess the effect of integrating smoking cessation therapy at OAT clinics compared with standard OAT (control arm) on self-reported number of cigarettes smoked and carbon monoxide levels in the exhaled air.

The secondary objectives are to investigate the change in psychological distress, impact of smoking cessation on inflammation, physical tests, and assessment of changes in quality of life, fatigue, and psychological well-being in the trial arms.

Trial design {8}

This study is designed as a multicentre individually randomised controlled superiority trial with two parallel groups and an allocation ratio of 1:1.

Methods: Participants, interventions and outcomes

Study setting {9}

The target group will be patients with severe opioid dependence receiving OAT from outpatient clinics in the Norwegian cities Bergen and Stavanger, who are smoking tobacco. The Department of Addiction Medicine at Haukeland University Hospital in Bergen and the Department of Substance Abuse and Addiction Treatment, Stavanger University Hospital in Stavanger have adopted an integrated treatment and care model for patients receiving OAT. In Bergen, OAT outpatient clinics have been established in each district where the patients are followed up by health and social workers on a nearly daily basis with observed intake of the OAT medications [2]. This group of patients has a large morbidity burden, and have to a limited degree been able to access other standard health care. Each of the OAT outpatient clinics is staffed by a consultant and a physician specialising in addiction medicine in addition to nurses, social workers, and several of the clinics also being staffed by a

psychologist. OAT Stavanger has a relatively similar structure. The treatment model in Bergen and Stavanger is an excellent platform to test out integration of additional intervention for patients receiving OAT aiming to improve health and life span of a vulnerable group, and at the same time gathering knowledge which traditionally have been very difficult to obtain.

Eligibility criteria {10}

For the randomised trial, inclusion will be based on the following criteria

- Receiving OAT from an included outpatient clinic with weekly follow-up
- Smoking at least one cigarette per day or seven cigarettes per week
- Obtaining informed consent

The following exclusion criteria will be used:

- Allergies or prior anaphylactic reactions to medication used
- Smoking less than three times a week
- Already using smoking cessation medications

Who will take informed consent? {26a}

Research nurses at the involved OAT clinics will recruit patients and obtain informed consent.

Additional consent provisions for collection and use of participant data and biological specimens {26b}

The consent forms specifically state that the collection of blood samples and physical tests are a part of the study and that participation is voluntary. Refusal to participate will involve no penalty or loss of benefits, and the subject may discontinue participation at any time without penalty or loss of benefits to which the subject is otherwise entitled. The specimens will only be used for research that is described in the protocol and consent. All biological specimens will be destroyed at the end of the study or at a specified time (e.g., after analysis). Patients are, in addition, asked to consent to the collection and storage of their specimens in a biobank connected to Bergen Addiction Research Group which is administered by the Department of Addiction medicine at the Haukeland University Hospital in Bergen, Norway.

Interventions

Explanation for the choice of comparators {6b}

Comparators and participants in the intervention group are recruited from the same involved OAT clinics, to reduce selection bias.

Intervention description {11a}

Participants randomised to the intervention arm will be assessed at baseline (Table 1) and offered a weekly smoking cessation intervention consisting of behavioural interventions for smoking cessation and prescription-free nicotine replacement products in addition to standard opioid replacement therapy. The behavioural intervention is based on motivational interviewing and psychoeducational techniques and will be delivered weekly during the intervention.

In addition to the behavioural intervention, participants will have the opportunity to pick up prescription free medications at the weekly appointments. Available medications are:

Nicotine patches (21 mg/24h, 14 mg/24h or 7 mg/24h), Nicotine lozenges or chewing gum (both in 1 mg or 2 mg per units). Figure 1 and Table 2 gives the exact dosing of the medication.

If participants have managed to reduce the number of smoked cigarettes per day by at least 50 % at week 16, they may extend the intervention for another eight weeks to attempt smoking cessation.

Participants randomized to the control arm will receive standard OAT treatment. At baseline, they will complete the assessment also offered to the intervention group (table 1).

Criteria for discontinuing or modifying allocated interventions {11b}

Participants will be allowed to change their choice of NRT medication, within the limits described in Figure 1, during the trial.

Strategies to improve adherence to interventions {11c}

Intervention will be linked to other treatment follow up to improve adherence.

Relevant concomitant care permitted or prohibited during the trial {11d}

There are no restrictions.

Provisions for post-trial care {30}

Following the trial participants will be offered yearly health assessments at their local OAT-clinic.

Outcomes {12}

Primary outcome measures are:

- Proportions of participants achieving smoking cessation verified by carbon monoxide (CO)-levels below six parts per million (ppm) at the end of the intervention or at least a 50% reduction in number of cigarettes smoked by week 16 of the intervention period (range 12-16 weeks after intervention initiation).

Secondary outcome measures are measured at the same time point as the primary outcome and include:

- Number of cigarettes smoked and CO-levels (in ppm) in exhaled air.
- Biochemical indicators of inflammation
    - All: C-reactive protein in serum and total leukocyte count in blood
    - Sub-group (n=60): IFN-gamma, IL-1beta, IL-1RA, IL-6, IL-8, IL-10, IL-17A, MCP-1, TNF-alfa measured in dried blood spots
- Changes in psychological well-being will be assessed with the Norwegian validated translation ten item version of Hopkins Symptom Checklist (SCL-10) [22], compared to baseline.
- Physical fitness assessed with 4-minute step-test measuring number of steps climbed in period [23].
- Changes in quality of life will be assessed with EuroQoL EQ-5D-5L [24], compared to baseline.
- Changes in fatigue will be assessed with the Fatigue Symptom Scale (FSS-3) [25], compared to baseline.
- Symptoms of dyspnoea will be assessed with the modified Medical Research Council dyspnoea scale (mMRC) [26].
- Physical activity is recorded with the International Physical Activity Questionnaire (IPAQ) [27].

Participant timeline {13}

Table 1

Sample size {14}

Smoking cessation:

We expect, based on a pilot study (not yet published), that the intervention will increase the proportion of patients not smoking by 11%.

The power calculation is based on the following assumptions:

- The power is set at 80% with a two-sided alpha (α) error of 5%
- Comparison of proportion of smokers at baseline and at 16 (12-16) weeks
- Up to 20% lost to follow-up at 16 weeks after treatment
- Equal proportions between the groups, 11% higher rates of smoking cessation compared to standard treatment and 4 % effect of comparison on smoking cessation in control arm.

Based on these assumptions, 133 persons are required in intervention arm and 133 persons in the control arm (statistical power calculations in Stata SE 17.0).

We also expect that the intervention will reduce the number of cigarettes smoked after the intervention by 30% if the patients do not achieve smoking cessation. Similarly, an additional power calculation is based on the following assumptions:

- The power is set at 90% with a two-sided alpha (α) error of 5%
- Comparison of proportion of smokers at baseline and at 16 (12-16) weeks
- Up to 20% lost to follow-up at 12 weeks after treatment
- Equal proportions between the groups, 30% reduction in cigarettes smoked compared to standard treatment in control arm.

Based on these assumptions, 133 persons are required in intervention arm and 133 persons in the control arm (statistical power calculations in Stata SE 17.0).

Recruitment {15}

All patients receiving OAT from included clinics will be considered the reference target population. As part of an annual health assessment linked to the ATLAS4LAR project [15], patients will be informed about the study and asked for consent to participation. All patients in target population will be offered annual health assessment and study participation. For those giving informed consent, an extended clinical assessment will be offered and those fulfilling inclusion and not exclusion criteria will be randomised for the study.

Assignment of interventions: allocation

Sequence generation {16a}

We will use computer generated block randomisation with a 1:1 ratio using blocks of eight to ensure relatively similar distribution between both arms throughout different time periods of the trial. Randomisation will be electronically registered. The randomisation will be stratified by site (site 1: Bergen and site 2: Stavanger).

Concealment mechanism {16b}

Once all eligibility criteria are fulfilled and consent is obtained a unique patient identifier number will be entered into the randomization spreadsheet generating the allocation to intervention or control arm.

Implementation {16c}

The allocation sequence is generated using a randomization algorithm made through Stata that is linked to an electronic number for each patient in a software for digital patient involvement. Research nurses will enrol and assign participants.

Assignment of interventions: Blinding

Who will be blinded {17a}

Even though complete blinding is regarded as difficult and infeasible. Patients will be informed of the follow-up they will receive, but not on other follow-up alternatives that are used or the exact hypotheses for the study. The outcomes assessor will be blinded.

Procedure for unblinding if needed {17b}

Not applicable, as study nurses know patient assignment.

Data collection and management

Plans for assessment and collection of outcomes {18a}

Data collection and follow-up will be given in line with *Table 1* and *Figure 1*.

The blood samples for the primary outcome measures will be collected at the OAT clinics. Outcome measures will be measured/collected at the OAT clinics by research nurses through a structured interview for both participants randomised to standard and integrated treatment. Following the intervention period, participants will receive a yearly health assessment as part of their standard OAT.

Plans to promote participant retention and complete follow-up {18b}

All participants who agree to participate in the trial will be shown appreciation for their willingness to participate. Undergoing the assessments the participants will be able to establish a relationship to research staff, which will provide a good opportunity for information and communication about issues concerning the trial. We will strive to give the trial participation experience a sense of being part of a community of patients taking part in research. Patients who discontinue will be offered a consultation without obligations with the study nurse to explore reasons for discontinuing. If participants discontinue the trial, they will be offered a new assessment at the time of the yearly health assessment. As the trial is integrated into ordinary OAT, patients will receive weekly follow-up and reminders of appointments with research staff.

Data management {19}

All data will be collected using electronic data collection software (Checkware®) under research nurses' supervision. Data is stored on a secure research server provided by the University Hospital of Bergen. All the clinical data, including information regarding OAT, OAT medication, substance use, and possible comorbid clinical conditions, will be collected from the electronic medical record.

Confidentiality {27}

All personal data is stored on a secure, access restricted research server. The senior investigators LTF and JHV will import data from the collection software (Checkware®) and from the electronic medical record to a common file using each participant's Norwegian personal identification number. Each participant is then given a computer-generated identification number for further analysis. Only anonymized data will be published.

Research nurses use paper forms for collecting the data during the trial and before data is plotted into the collection software. Appointments are made using the medical record system. The research nurses store all paper forms that may be connected to a participant in a locked file in a room with restricted access.

For documentation and follow-up purposes data will be stored until the end of the project on the 31th of December 2029, and then deleted.

Plans for collection, laboratory evaluation and storage of biological specimens for genetic or molecular analysis in this trial/future use {33}

Biological specimens collected in this trial are blood samples. These will be analysed and only the results will be stored. For patients who consent to collection of specimens for the biobank, the specimens will be stored in the biobank (ethical approval 2016/1080/REK vest). According to the ethical approval use of the samples in the biobank for future projects requires new ethical review.

Statistical methods

Statistical methods for primary and secondary outcomes {20a}

A detailed plan for analysis is provided in Table 3. Analysis methods will follow the CONSORT and SPIRIT guidelines [28-30]. All tests will be two-sided. Descriptive results and efficacy estimates will be presented with 95% confidence intervals. The statistical significance is set at $p < 0.05$. Potential confounders may be considered for adjustment if they are imbalanced at baseline (with assumed meaningful differences). variables will be summarized as percentages and continuous variables as medians with interquartile ranges or means with standard deviation for variables with a Gaussian

distribution. The main outcomes will be analysed with generalized linear models (Gaussian distribution).

Interim analyses {21b}

Throughout the study period there will be weekly meeting between the study nurses and the investigators. The meeting will consider progression of the trial and adverse events. The principal investigator LTF will make the final decision to terminate the trial.

Methods for additional analyses (e.g. subgroup analyses) {20b}

See Table 3

Methods in analysis to handle protocol non-adherence and any statistical methods to handle missing data {20c}

We will use an intention to treat analysis strategy to handle protocol non-adherence. Missing data will be considered, and appropriate imputations based on pre-defined assumptions will be done when necessary (as described in detailed plan of analysis).**Plans to give access to the full protocol, participant level-data and statistical code {31c}**

Upon request the access to the full protocol, anonymized participant-level dataset and statistical code is granted.

Oversight and monitoring

Composition of the coordinating centre and trial steering committee {5d}

Research nurses and the primary investigator (medical doctor) will meet on a weekly basis, during the study. The clinical team, including research nurse, at each OAT clinic will meet on a daily basis. Once a week the principal investigator, other investigators, research nurses and user representatives will meet (study coordination unit).

Composition of the data monitoring committee, its role and reporting structure {21a}

An external data monitoring committee is not needed as this study is a health services research evaluating the integration of a smoking cessation intervention. The medications used in the study are used according to the manufacturers' recommendation, and evaluating the effect of the medications is not a primary objective outcome of this study but rather an integrated smoking cessation intervention package.

Adverse event reporting and harms {22}

Those who participate in the study will be randomised to one of two different follow-up programs. It is possible that the intervention will be inferior in outcomes compared to the standard treatment. Some might have allergies to components in the administered nicotine products, but severe allergies to these are rare, and patients with severe allergies who are vulnerable to negative reactions will be excluded from participation in this trial. For nicotine gum products hiccoughs, gastrointestinal disturbances, jaw pain, and oro-dental problems are the most frequently reported side effects [31]. Nicotine lozenges have been reported to cause hiccoughs, burning and smarting sensation in the mouth, sore throat, coughing, dry lips, and mouth ulcers, whereas skin sensitivity and local skin irritation is common among users of nicotine patches [31]. Adverse events or unintended effects may be reported to the research nurses at daily meetings in the OAT clinics. Patients reporting adverse events will be offered an evaluation by the research nurse or research physician. The study coordination unit will be responsible to ensure safety, adherence to the protocol, quality of the study, and ethical conduct. It is also possible that follow-up with motivational interviewing approach could be considered as time consuming and unwanted. The participants could at any time choose to abort this intervention.

Frequency and plans for auditing trial conduct {23}

Bi-annual internal auditing of trial will be conducted.

Plans for communicating important protocol amendments to relevant parties (e.g. trial participants, ethical committees) {25}

Any substantial modifications to the protocol, which may impact on the conduct of the study, will be reported to the regional ethical committee for renewed approval. Participants will be informed of the changes and the published protocol will be amended accordingly.

Dissemination plans {31a}

Results of the trial will be published in peer-reviewed medical journals. We will submit abstracts to relevant national and international congresses. Participants and clinical staff at the participating OAT clinics will receive summaries of the outcomes.

Discussion

This study will improve understanding of how a combined intervention aiming for smoking cessation over a period of up to six months could succeed in reducing smoking, and improve psychological well-being and physical tests. This could potentially reduce future morbidity and mortality among patients with opioid dependence receiving OAT.

Smoking cessation or reduction has been considered as infeasible for most patients receiving OAT [13]. Studies have investigated interventions to quit smoking among patients with opioid dependence [16,21,32], but few have investigated smoking cessation interventions integrated into an OAT setting. Although OAT patients are a hard-to-reach group of patients, a previous study has shown that integrating treatment into the ordinary OAT follow-up increases adherence and completion rates [33]. We believe this study will be able to answer some of the questions raised in a recent meta-analysis on reducing tobacco use in people experiencing homelessness [34], including increasing accessibility, long-time follow-up impact on mental health, and substance use.

Our trial involves some limitations and several strengths. For the trial, it is difficult to ensure complete blinding. The study is funded from public sources ensuring independency. We also have a biologically verified primary outcome. Thus, substantial information biases are considered unlikely. The study is individually randomised minimising potential confounding. The study population receiving OAT will include a sufficient large study sample size to answer the primary objectives with high precision and is assumed to have adequate precision also for secondary objectives. In terms of safety, the trial is considered as a low-risk study. Our conventional trial design is less vulnerable to confounding from time trends than other designs such as a stepped-wedge design.

If the smoking cessation intervention integrated into OAT is superior to standard OAT in terms of assessed outcomes, this intervention could be considered for further scale-up.

Trial status

Trial protocol version 2, 14.07.2022. Start of recruitment 19.April 2022. Estimated completion of recruitment June 19. 2022.

Abbreviations

COPD: Chronic Obstructive Pulmonary Disease

OAT: Opioid Agonist Therapy

NRT: Nicotine Replacement Therapy

CO: Carbon Monoxide

SCL-10: Hopkins Symptom Checklist

mMRC: modified Medical Research Council dyspnoea scale

IPAQ: International Physical Acitvity Questionaire

CRP: C-Reactive Protein

FSS-3: Fatigue Symptom Scale

PPM: parts per million.

Declarations

Acknowledgements

We thank the devoted clinical staff for their enthusiasm during the planning of the project as well as involved participants. We also thank all participating investigators in ATLAS4LAR including:

Bergen: Vibeke Bråthen Buljovcic, Jan Tore Daltveit, Karl Trygve Druckrey-Fiskaaen, Lars T. Fadnes (principal investigator), Trude Fondenes, Per Gundersen, Siv-Elin Leirvåg Carlsen, Mette Hegland Nordbotn, Maria Olsvold, Marianne Cook Pierron, Christine Sundal, and Jørn Henrik Vold.

Stavanger: Maren Borsheim Bergsaker, Eivin Dahl, Tone Lise Eielsen, Torhild Fiskå, Einar Furulund, Torgeir Gilje Lid, Eirik Holder, Tesfaye Madebo, Tine Selmer Cruickshank, and Ewa Joanna Wilk.

Authors' contributions {31b}

All authors (KTDF, EF, JTD, JHV, TGL, TM, LTF) have been involved in design of the study, contributed to implementation, and writing of the protocol. KTDF wrote the first draft and led the design process. All authors have read and approved the final manuscript

Funding {4}

The study was funded by Western Norway Regional Health Authority («*Strategiske forskningsmidler*» through ATLAS4LAR-project) with Department of Addiction Medicine, Haukeland University Hospital as responsible institution. The funders had no role in study design, data collection and analysis, decision to publish, or preparation of the manuscript.

Availability of data and materials {29}

Authors and persons mentioned under acknowledgements will have access to the final trial dataset.

Ethics approval and consent to participate {24}

Except from use of a few hours of time from the participants and some examinations such as blood sample collection can be regarded as unpleasant, participation is not believed to be linked with substantial risks. The study has been approved by regional ethical committee (no. 155386/ REK Sør-øst -B, dated 23.09.2020/ 03.12.2021/ 05.04.2022). The trial will be conducted in strict accordance with the Declaration of Helsinki and other international conventions and with good clinical practice and good laboratory practice[35,36]. Written informed consent and assent will be obtained from each participant.

Consent for publication {32}

Not applicable - no identifying images or other personal or clinical details of participants are presented here or will be presented in reports of the trial results. Informed consent materials are available from the corresponding author on request."

Competing interests {28}

The authors have no competing interests.

Authors' information (optional)

KTDF is a consultant in addiction medicine at the Haukeland University hospital and a PhD fellow at the University of Bergen.

References

1. Forouzanfar MH, Alexander L, Anderson HR, Bachman VF, Biryukov S, Brauer M, Burnett R, Casey D, Coates MM, Cohen A *et al*: **Global, regional, and national comparative risk assessment of 79 behavioural, environmental and occupational, and metabolic risks or clusters of risks in 188 countries, 1990-2013: a systematic analysis for the Global Burden of Disease Study 2013**. *Lancet* 2015, **386**(10010):2287-2323.

2. Collaborators GBDCoD: **Global, regional, and national age-sex-specific mortality for 282 causes of death in 195 countries and territories, 1980-2017: a systematic analysis for the Global Burden of Disease Study 2017**. *Lancet* 2018, **392**(10159):1736-1788.
3. Inoue-Choi M, Christensen CH, Rostron BL, Cosgrove CM, Reyes-Guzman C, Apelberg B, Freedman ND: **Dose-Response Association of Low-Intensity and Nondaily Smoking With Mortality in the United States**. *JAMA Netw Open* 2020, **3**(6):e206436.
4. Inoue-Choi M, Hartge P, Park Y, Abnet CC, Freedman ND: **Association Between Reductions of Number of Cigarettes Smoked per Day and Mortality Among Older Adults in the United States**. *Am J Epidemiol* 2019, **188**(2):363-371.
5. Hayes RD, Chang CK, Fernandes A, Broadbent M, Lee W, Hotopf M, Stewart R: **Associations between substance use disorder sub-groups, life expectancy and all-cause mortality in a large British specialist mental healthcare service**. *Drug Alcohol Depend* 2011, **118**(1):56-61.
6. Lewer D, Jones NR, Hickman M, Nielsen S, Degenhardt L: **Life expectancy of people who are dependent on opioids: A cohort study in New South Wales, Australia**. *J Psychiatr Res* 2020, **130**:435-440.
7. Rehm J, Manthey J, Shield KD, Ferreira-Borges C: **Trends in substance use and in the attributable burden of disease and mortality in the WHO European Region, 2010-16**. *Eur J Public Health* 2019, **29**(4):723-728.
8. Westman J, Wahlbeck K, Laursen TM, Gissler M, Nordentoft M, Hällgren J, Arffman M, Ösby U: **Mortality and life expectancy of people with alcohol use disorder in Denmark, Finland and Sweden**. *Acta Psychiatr Scand* 2015, **131**(4):297-306.
9. Aas CF, Vold JH, Skurtveit S, Lim AG, Ruths S, Islam K, Askildsen JE, Løberg EM, Fadnes LT, Johansson KA: **Health-related quality of life of long-term patients receiving opioid agonist therapy: a nested prospective cohort study in Norway**. *Subst Abuse Treat Prev Policy* 2020, **15**(1):68.
10. Guydish J, Passalacqua E, Pagano A, Martinez C, Le T, Chun J, Tajima B, Docto L, Garina D, Delucchi K: **An international systematic review of smoking prevalence in addiction treatment**. *Addiction* 2016, **111**(2):220-230.
11. Bech AB, Clausen T, Waal H, Delaveris GJM, Skeie I: **Organ pathologies detected post-mortem in patients receiving opioid agonist treatment for opioid use disorder: a nation-wide 2-year cross-sectional study**. *Addiction* 2022, **117**(4):977-985.
12. Mehta S, Parmar N, Kelleher M, Jolley CJ, White P, Durbaba S, Ashworth M: **COPD and asthma in patients with opioid dependency: a cross-sectional study in primary care**. *NPJ Prim Care Respir Med* 2020, **30**(1):4.
13. Apollonio D, Philipps R, Bero L: **Interventions for tobacco use cessation in people in treatment for or recovery from substance use disorders**. *Cochrane Database Syst Rev* 2016, **11**:CD010274.
14. Guydish J, Kapiteni K, Le T, Campbell B, Pinsker E, Delucchi K: **Tobacco use and tobacco services in California substance use treatment programs**. *Drug Alcohol Depend* 2020, **214**:108173.
15. Vlad C, Arnsten JH, Nahvi S: **Achieving Smoking Cessation Among Persons with Opioid Use Disorder**. *CNS Drugs* 2020, **34**(4):367-387.
16. Yee A, Hoong MC, Joyce YC, Loh HS: **Smoking Cessation Among Methadone-Maintained Patients: A Meta-Analysis**. *Subst Use Misuse* 2018, **53**(2):276-285.

17. Notley C, Gentry S, Livingstone-Banks J, Bauld L, Perera R, Hartmann-Boyce J: **Incentives for smoking cessation**. *Cochrane Database Syst Rev* 2019, **7**(7):CD004307.
18. Lindson-Hawley N, Thompson TP, Begh R: **Motivational interviewing for smoking cessation**. *Cochrane Database Syst Rev* 2015(3):CD006936.
19. Patnode CD, Henderson JT, Coppola EL, Melnikow J, Durbin S, Thomas RG: **Interventions for Tobacco Cessation in Adults, Including Pregnant Persons: Updated Evidence Report and Systematic Review for the US Preventive Services Task Force**. *Jama* 2021, **325**(3):280-298.
20. Park ER, Perez GK, Regan S, Muzikansky A, Levy DE, Temel JS, Rigotti NA, Pirl WF, Irwin KE, Partridge AH *et al*: **Effect of Sustained Smoking Cessation Counseling and Provision of Medication vs Shorter-term Counseling and Medication Advice on Smoking Abstinence in Patients Recently Diagnosed With Cancer: A Randomized Clinical Trial**. *Jama* 2020, **324**(14):1406-1418.
21. Hall SM, Humfleet GL, Gasper JJ, Delucchi KL, Hersh DF, Guydish JR: **Cigarette Smoking Cessation Intervention for Buprenorphine Treatment Patients**. *Nicotine Tob Res* 2018, **20**(5):628-635.
22. Strand BH, Dalgard OS, Tambs K, Rognerud M: **Measuring the mental health status of the Norwegian population: a comparison of the instruments SCL-25, SCL-10, SCL-5 and MHI-5 (SF-36)**. *Nord J Psychiatry* 2003, **57**(2):113-118.
23. Vieira EB, Degani-Costa LH, Amorim BC, Oliveira LB, Miranda-Silva T, Sperandio PC, Medeiros WM, Arbex FF, Ramos RP, Nery LE: **Modified BODE Index to Predict Mortality in Individuals With COPD: The Role of 4-Min Step Test**. *Respir Care* 2020, **65**(7):977-983.
24. Peak J, Goranitis I, Day E, Copello A, Freemantle N, Frew E: **Predicting health-related quality of life (EQ-5D-5 L) and capability wellbeing (ICECAP-A) in the context of opiate dependence using routine clinical outcome measures: CORE-OM, LDQ and TOP**. *Health Qual Life Outcomes* 2018, **16**(1):106.
25. Vold JH, Gjestad R, Aas CF, Meland E, Johansson KA, Fadnes LT: **Validation of a three-item Fatigue Severity Scale for patients with substance use disorder: a cohort study from Norway for the period 2016-2020**. *Health Qual Life Outcomes* 2021, **19**(1):69.
26. Hajiro T, Nishimura K, Tsukino M, Ikeda A, Koyama H, Izumi T: **Analysis of clinical methods used to evaluate dyspnea in patients with chronic obstructive pulmonary disease**. *Am J Respir Crit Care Med* 1998, **158**(4):1185-1189.
27. Craig CL, Marshall AL, Sjöström M, Bauman AE, Booth ML, Ainsworth BE, Pratt M, Ekelund U, Yngve A, Sallis JF *et al*: **International physical activity questionnaire: 12-country reliability and validity**. *Med Sci Sports Exerc* 2003, **35**(8):1381-1395.
28. Calvert M, Kyte D, Mercieca-Bebber R, Slade A, Chan AW, King MT, Hunn A, Bottomley A, Regnault A, Chan AW *et al*: **Guidelines for Inclusion of Patient-Reported Outcomes in Clinical Trial Protocols: The SPIRIT-PRO Extension**. *Jama* 2018, **319**(5):483-494.
29. Welch VA, Norheim OF, Jull J, Cookson R, Sommerfelt H, Tugwell P: **CONSORT-Equity 2017 extension and elaboration for better reporting of health equity in randomised trials**. *Bmj* 2017, **359**:j5085.

30. Moher D, Schulz KF, Altman DG: **The CONSORT statement: revised recommendations for improving the quality of reports of parallel-group randomised trials.** *Lancet* 2001, **357**(9263):1191-1194.
31. Hartmann-Boyce J, Chepkin SC, Ye W, Bullen C, Lancaster T: **Nicotine replacement therapy versus control for smoking cessation**. *Cochrane Database Syst Rev* 2018, **5**(5):Cd000146.
32. Nahvi S, Adams TR, Ning Y, Zhang C, Arnsten JH: **Effect of varenicline directly observed therapy versus varenicline self-administered therapy on varenicline adherence and smoking cessation in methadone-maintained smokers: a randomized controlled trial.** *Addiction* 2021, **116**(4):902-913.
33. Fadnes LT, Aas CF, Vold JH, Leiva RA, Ohldieck C, Chalabianloo F, Skurtveit S, Lygren OJ, Dalgård O, Vickerman P *et al*: **Integrated treatment of hepatitis C virus infection among people who inject drugs: A multicenter randomized controlled trial (INTRO-HCV)**. *PLoS Med* 2021, **18**(6):e1003653.
34. Vijayaraghavan M, Elser H, Frazer K, Lindson N, Apollonio D: **Interventions to reduce tobacco use in people experiencing homelessness**. *Cochrane Database Syst Rev* 2020, **12**(12):CD013413.
35. European Medicines Agency: **Guideline for good clinical practice E6(R2)** 6 edn. 2018. https://www.ema.europa.eu/en/documents/scientific-guideline/ich-e-6-r2-guideline-good-clinical-practice-step-5_en.pdf Accessed 01. May 2022
36. ENVIRONMENT DIRECTORATE CHEMICALS GROUP AND MANAGEMENT COMMITTEE: **OECD Principles on Good Laboratory Practice**. 1998 https://www.oecd.org/officialdocuments/publicdisplaydocumentpdf/?cote=env/mc/chem(98)17&doclanguage=en. Accessed 01. May 2022

Table 1: Protocol schedule outlining follow-up visits and assessments at each visit

	STUDY PERIOD					
	Enrolment	Allocation	Post-allocation			Follow-up
**TIME POINT**	**-1**	**0**	**1 - 15**	**16 (12-20)**	**17 - 24**	**34 - 54**
**Research nurse assessment**	X			X		X
**Informed consent**	X					
**Eligibility assessment**	X					
**Randomization**		x				
**Weekly follow-up by OAT staff (intervention group)**			x		x	
**Clinical assessment**	X			X		X
**CO-measurement**	X			X		X
**Physical fitn.. (4-min step-test)**	X			X		X
**Full blood count, CRP***	X			X		X

**SCL-10 (mental health)**	X	X	X
**FSS-3 (fatigue symptoms)**	X	X	X
**EQ-5D-5L (quality of life)**	X	X	X

* Additional tests will be taken on clinical indication. Time points indicate weeks from initiation of intervention.

Table 2: Dosing table for nicotine chewing gum and lozenges.

	**Recommended daily dose**	**Maximum daily dose**
**Patch and 2 mg chewing gum combined**	1 patch<br>6 chewing gums	1 patch<br>24 chewing gums
**Patch and 1 mg lozenges combined**	1 patch<br>6 lozenges	1 patch<br>24 lozenges
**Patch and 2 mg lozenges combined**	1 patch<br>6 lozenges	1 patch<br>15 lozenges
**Chewing gum (2 mg) monotherapy**	6 chewing gums	24 chewing gums
**Lozenges (1 mg) monotherapy**	6 lozenges	24 lozenges
**Lozenges (2 mg) monotherapy**	6 lozenges	15 lozenges

The table shows possible doses of nicotine chewing gum and lozenges as monotherapy or combined with nicotine patches. Patches are available in doses of 7 mg, 14 mg or 21 mg per 24 hours.

Table 3: Analysis plan

Variable/ outcome	Hypothesis	Outcome measure	Method of analysis
**1. Primary**			

a.	Proportion of patients smoking	Intervention improves smoking cessation rates from baseline to 16 weeks	Carbon monoxide in ppm in exhaled air	Chi-squared test
b.	Proportion achieving at least 50 % reduction in number of cigarettes smoked	Intervention reduces number of cigarettes smoked from baseline to 16 weeks	Self reported daily number of cigarettes smoked	Chi-squared test

2. Secondary

Number of cigarettes smoked	Reduction in number of cigarettes	Self reported daily number of cigarettes smoked	t-test and regression methods with secondary outcomes as dependent
Carbon monoxide in exhaled air	Reduced CO-levels	Carbon monoxide in ppm in exhaled air	variable adjusted for variables defined under
C-reactive protein	Reduced levels	CRP in mg/L	additional analysis
Leucocyte count	Levels within reference limit	Leucocyte count in 10^9/L	
Psychological well-being	Increased score	Hopkins Symptom Checklist (SCL-10)	
Physical fitness	Increased score	4-minute step test, number of steps	
Quality of life	Increased score	EuroQoL EQ-5D-5L-questionaire	
Fatigue	Less Fatigue	Fatigue Symptom Scale (FSS-3)	
Dyspnoea	Less after intervention	modified Medical Research Council (mMRC)-scale	
Physical activity	increased	Physical Activity Questionnaire (IPAQ)	

3. Additional analysis

OAT-medication	Choice of OAT-medication impacts primary outcome	Regression methods with OAT medication as categorical co-variate.
OAT-medication doses	Higher doses inhibits smoking cessation	Regression methods with OAT-doses as independent variable
Adjusted for age<br>Adjusted for sex<br>Adjusted for i.v. drug use<br>Adjusted for known COPD	Co-variates impact the outcomes of the trial	Regression methods with appropriate interaction term
Impact of number of cigarettes smoked on secondary outcomes	Fewer cigarettes smoked results in improved secondary outcomes	Regression methods with secondary outcome as dependent variable and number of cigarettes smoked as independent variable

Important remarks:

In all analyses will be expressed as coefficient, standard errors, corresponding 95% and associated p-values
Goodness-of-fit will be assessed by examining the residuals for model assumptions and chi-squared test of goodness-of-fit.
Bonferroni method will be used to correct for multiple testing

Figure 1: Timeline for nicotine replacement therapy component of the smoking cessation intervention.

Remarks: The blue columns indicate evaluation points.

Update of statistical analysis plan for: Integration of smoking cessation into standard treatment for patients receiving opioid agonist therapy who are smoking tobacco: protocol for a randomised controlled trial (ATLAS4LAR)

Abstract:

This protocol paper presents an updated statistical analysis plan of the protocol of a randomised controlled trial. The randomised controlled trial investigates the effect of integrating smoking cessation interventions at outpatient opioid agonist therapy (OAT) clinics for persons with opioid dependency receiving OAT medication. The intervention group receives weekly follow-up including a short behavioural intervention and provision of nicotine replacement products. The control group receives standard treatment. The duration of the intervention is 16 weeks and the follow-up was completed by the end of October 2023. The primary outcome is defined as the proportion of participants reducing the number of cigarettes smoked by at least a 50% at week 16 of the intervention period. The primary outcome will be analysed according to intention-to-treat principles. Missing outcome data will be set equal to the baseline values development and reporting of the statistical analysis plan follow the Guidelines for the Content of Statistical Analysis Plans in Clinical Trials.

Trial registration

ClinicalTrials.gov NCT05290025. Registered on 22 March 2022

Administrative information

SAP version number with dates	Version 2, 27.09.2023
Reference to version of protocol being used	Version 2, 14.07.2022
SAP revision history	Version 1 provided in protocol from 14.07.2022.
Justification for each SAP revision	Version 2 contains more detail to comply with SAP checklist [37]
Timing of SAP revisions in relation to interim analyses, etc.	No interim analysis completed. Version 2 of SAP published ahead of completion of follow-up
Names, affiliations, and roles of SAP contributors	Karl Trygve Druckrey-Fiskaaen[1,2*], Tesfaye Madebo[1,3,6],Jan Tore Daltveit[1], Jørn Henrik Vold[1,2,4], Einar Furulund[1,2,5],Torgeir Gilje Lid[5,7], Lars Thore Fadnes[1,2]<br>[1] Bergen Addiction Research, Department of Addiction Medicine, Haukeland University Hospital, Bergen, Norway<br>[2] Department of Global Public Health and Primary Care, University of Bergen, Norway<br>[3] Department of Respiratory Medicine, Stavanger University Hospital, Stavanger, Norway<br>[4] Division of Psychiatry, Haukeland University Hospital, Bergen, Norway.<br>[5] Centre for Alcohol and Drug Research, Stavanger University Hospital, Stavanger, Norway<br>[6] Department of Clinical Science, University of Bergen, Norway

	[7] Department of Public Health, University of Stavanger, Norway
Person writing the SAP	Karl Trygve Druckrey-Fiskaaen
Senior statistician/ analyst responsible	Jørn Henrik Vold
Chief investigator/clinical lead	Lars Thore Fadnes

Introduction

Background and rationale

About 85% of patients receiving opioid agonist therapy (OAT) for opioid dependence smoke tobacco [10]. Although smoke-related pulmonary diseases are significant contributors to morbidity and mortality, few smoking cessation interventions are evaluated within this group [15], and few OAT patients are offered smoking cessation as an integrated part of their addiction treatment[38]. The integration of hepatitis C virus treatment at OAT clinics improved the time to treatment initiation and the rate of sustained virological response [33]. This trial aimed to investigate whether a similar effect can be seen for the integration of smoking cessation therapy [39]. More specifically the trial aims to investigate the effect of a combined smoking cessation intervention administered weekly for up to 16 weeks on smoking patterns, psychological well-being, and physical tests. See the protocol article for further detail [39].

Objectives

The primary objective is to assess the effect of integrating smoking cessation therapy at OAT clinics compared with standard OAT (control arm). Smoking cessation is measured by carbon monoxide levels in the exhaled air and the self-reported number of cigarettes smoked.

The secondary objectives are to investigate the change in psychological distress, impact of smoking cessation on inflammation, physical tests, and assessment of changes in quality of life, fatigue, and psychological well-being in the trial arms. The secondary objectives are specified in more detail in the published study protocol [39].

The study protocol for the randomised controlled trial on integrated smoking cessation treatment for patients who receive OAT was published in August 2022 [39]. The process of recruitment and inclusion lasted longer than anticipated at the time of publication. Recruitment was completed in July 2023. Follow-up was completed by the end of October 2023. In preparing for the analysis and publication of the primary outcomes of the trial there was a need to update and expand the statistical analysis plan included in the protocol [39]. We have used the Guidelines for the Content of Statistical Analysis Plans in Clinical Trials to guide the update [37]. The Statistical Analysis Plan (SAP) Checklist v 1.0 2019 is provided in Supplementary File 1.

Study methods

The study is designed as a multicentre individually randomised controlled superiority trial with two parallel groups and an allocation ratio of 1:1.

The sample size calculations are provided in the protocol [39]. Based on the calculations 133 persons were required in intervention arm and 133 persons in the control arm.

No interim analyses were planned (see details in section 21b in the protocol [39]) and thus, no adjustments to significance levels were assessed.

The primary outcomes will be analysed collectively upon completion of follow-up.

Statistical principles

All tests will be two-sided. Descriptive results and efficacy estimates will be presented with 95% confidence intervals. The statistical significance was set at $p < 0.05$.

There is only one primary outcome and thus no need to correct for multiplicity.

Adherence is defined as at least 50% attendance at weekly appointments. The total duration possible is 16 weeks.

Adherence will be presented as a histogram of the proportion of participants in the intervention each week from 0-16. A supplementary table will provide the number of trial participants who attended which percentage of study visits.

According to Figure 1 of the protocol the participants were recommended a timeline for nicotine replacement therapy [39]. We will record deviations from the timeline for example not reducing the dose of nicotine replacement products according to the plan.

We will analyse the outcomes with an intention to treat and per protocol approach. See table 2 for more detail.

Trial population

Figure 1 shows the information to be included in the CONSORT flow-diagram.

The withdrawals/lost to follow up will be handled according to the following principles:

- The week and, if provided by the participant, the reason for withdrawal will be noted. According to the ethics approval (no. 155386/REK Sør-øst-B, dated 23 September 2020/03 December 2021/05 April 2022) participants are allowed to withdraw without giving reasons.
- A histogram will be produced showing the number of withdrawals and loss to follow-up for each week of the trial.
- The proportion of withdrawal and loss to follow-up for the trial in total will be calculated as the sum of withdrawals and loss to follow-up each week divided by the number of persons allocated to each arm of the trial.

Table 1 indicates the baseline characteristics and how they will be summarized.

Analysis

We will use Stata/SE17 (StataCorp, TX, USA) for the statistical analysis.

Primary outcome:

The analysis of the primary outcome measures will be completed according to intention to treat (ITT) principles (Table 2). If primary outcome data are missing, we will set these equal to baseline values for the ITT analysis. The primary outcome is defined as the proportion of participants who achieve at least a 50% reduction in the number of cigarettes smoked by week 16 of the intervention period (range 12–16 weeks after intervention initiation), including those who achieve smoking cessation. This is assessed with self-reported cigarette use and verified by carbon monoxide (CO) levels and expected to be below six parts per million (ppm) among self-reported non-smokers.

A sensitivity analysis of the effect of missing data will be completed according to Table 3.

The proportions of smokers in both arms at baseline and 16 weeks will be presented in a bar chart.

We will perform exploratory (hypothesis generating) subgroup analysis (presented as forest plots) of the primary outcome with the following subgroups (at baseline):

- Age: < 40, 40-60, > 60
- Sex (male/female)
- Obstructive pulmonary disease (yes/no)
- OAT medication (buprenorphine vs. methadone/other)
- Intravenous drug use (yes/no)
- Years of smoking: < 5, 5-15, > 15 (not in the original analysis plan)

Secondary outcome:
The secondary outcomes will be analysed according to Table 4 as changes from baseline (day of enrolment). Data for the secondary analysis will be collected by week 16 of the intervention (range 12–16 weeks after intervention initiation).

Additional analysis
We will examine the validity of self-reported cigarette use by correlation and/or Spearman's rank between the number of cigarettes smoked and smoking intensity determined by carbon monoxide in exhaled air [40].

Methods used for assumptions to be checked for statistical methods
The participants were randomly assigned to ensure comparable intervention and control arms. The analysis methods will follow the CONSORT and SPIRIT guidelines. Categorical or continuous variables will be summarized as percentages, median with interquartile range or means with standard deviation for variables with Gaussian distribution. The outcomes will be checked for the assumptions of independent outcomes, limited influence of outliers and non-multicollinearity hold.

Potential confounders may be considered for adjustment if they are imbalanced at baseline. Missing data will be considered, and imputation based on predefined assumptions (baseline values) will be performed when necessary.

Handling of missing data
Missing data in the outcome variables will be handled using an intention to treat strategy, i.e. the value is set equal to baseline.

Adverse event reporting and harms
We will report the number of grade 3/4 adverse events and for each event details of the event and causality considerations.

Table 1: Baseline characteristics and summary statistics

	Intervention	Control
Males n (%)		
Females n (%)		
Age median (IQR[a])		
Education < 10 years n (%)		
Education 10-12 years n (%)		
Education > 12 years n (%)		
Homelessness n (%)		
Social security benefits as income n (%)		
Formal work as income n (%)		
Methadone *n (%)*		
Buprenorphine *n (%)*		
Other opioid agonist treatment medications		
Substance use last 30 days n (%)		
Illicit opioids		
Alcohol		
Amphetamines or cocaine		
Benzodiazepines		
Cannabis		
I.v. drug use last 6 months		
Debut age smoking median (IQR[a])		
Years of smoking median (IQR[a])		
Average daily number of cigarettes smoked		
Body mass index median (IQR[a])		
Obstructive pulmonary disease[b] n (%)		

[a] Inter-quartile range

[b] Forced expiratory volume in the first second (FEV1)/ Forced vital capacity (FVC) ratio < 70 % in spirometry.

Table 2: Analysis and presentation of the primary outcome

Outcome	Events n (%)		Absolute difference between arms (n/%, 95 %CI)	Logistic Regression[a] Odds ratio (95 % CI)
	Intervention	Control		
Smokers[b] at 16 weeks, ITT[c]				
Carbon monoxide < 6 ppm, N (%)				
Smokers at 16 weeks, PP[d]				
At least 50 % reduction number[e] of cigarettes at 16 weeks, ITT[c]				
At least 50 % reduction number of cigarettes at 16 weeks, PP[d]				
Number of cigarettes smoked/day[c]				
Severe adverse events (assumed linked)				

[a]Unadjusted analysis unless Table 1 indicate substantial differences between arms at baseline.
[b]A person smoking at least one cigarette per day or seven cigarettes per week
[c]ITT = intention to treat population: Participants assessed according to randomisation regardless of adherence to trial. Any missing data in the outcome variable will be set equal to baseline.
[d]PP = per protocol population: All participants who completed at least 50% of the trial visits
[e] The average daily number of cigarettes smoked, as reported by the participant.

Table 3: Sensitivity analysis of handling missing data on the primary outcome

Outcome	Handling of missing data at end of trial	Events n (%)		Absolute difference between arms (n, 95 %CI)	Logistic Regression[a] Odds ratio (95 % CI)
		Intervention	Control		
No. of smokers[b] at 16 weeks, ITT[c]	Equal to baseline[d]				
	Person excluded[e]				
No. of smokers at 16 weeks, PP[f]	Equal to baseline[d]				
	Person excluded[e]				
At least 50 % reduction number[g] of cigarettes at 16 weeks, ITT	Equal to baseline[d]				
	Person excluded[e]				
At least 50 % reduction number of cigarettes at 16 weeks, PP	Equal to baseline[d]				
	Person excluded[e]				

[a] Unadjusted analysis unless Table 1 indicates substantial differences between arms at baseline.
[b] A person smoking at least one cigarette per day or seven cigarettes per week
[c] ITT = intention to treat population: Participants assessed according to randomisation regardless of adherence to trial.
[d] If data on primary outcome is missing at 16 weeks, the results are set equal to baseline.
[e] If data on primary outcome is missing at 16 weeks the person is excluded from the analysis (complete case).
[f] PP = per protocol population: All participants who completed at least 50% of the trial visits
[g] The average daily number of cigarettes smoked, as reported by the participant.

Table 4: Plan for analysis of secondary outcomes

Outcome	Hypothesis	Outcome measure	Method of analysis
Number of cigarettes smoked	Reduction in number of cigarettes	Self-reported daily number of cigarettes smoked	t-test and regression methods with secondary outcomes as dependent variable adjusted for variables defined in Figure 2
Carbon monoxide in exhaled air	Reduced CO levels	Carbon monoxide in ppm in exhaled air	
C-reactive protein	Reduced levels	CRP in mg/L	
Leucocyte count	Levels within reference limit	Leucocyte count in 10^9/L	
Psychological well-being	Increased score	Hopkins Symptom Checklist (SCL-10)	
Physical fitness	Increased score	4-min step test, number of steps	
Quality of life	Increased score	EuroQoL EQ-5D-5L-questionaire	
Fatigue	Less Fatigue	Fatigue Symptom Scale (FSS-3)	
Dyspnoea	Less after intervention	Modified Medical Research Council (mMRC)-scale	
Physical activity	Increased	Physical Activity Questionnaire (IPAQ)	

Figure 1: CONSORT Flow diagram

Declarations

Ethics approval and consent to participate

Except from use of a few hours of time from the participants and some examinations such as blood sample collection can be regarded as unpleasant, participation is not believed to be linked with substantial risks. The study has been approved by regional ethical committee (no. 155386/ REK Sør-øst -B, dated 23.09.2020/ 03.12.2021/ 05.04.2022). The trial will be conducted in strict accordance with the Declaration of Helsinki and other international conventions and with good clinical practice and good laboratory practice [35,36]. Written informed consent and assent will be obtained from each participant.

Consent for publication

Not applicable - no identifying images or other personal or clinical details of participants are presented here or will be presented in reports of the trial results. Informed consent materials are available from the corresponding author on request.

Availability of data and materials

Authors and persons mentioned under acknowledgements will have access to the final trial dataset.

Competing interests

The authors have no competing interests.

Funding

The study was funded by Western Norway Regional Health Authority («*Strategiske forskningsmidler*» through ATLAS4LAR-project) with Department of Addiction Medicine, Haukeland University Hospital as responsible institution. The funders had no role in study design, data collection and analysis, decision to publish, or preparation of the manuscript.

Authors' contributions

All authors (KTDF, EF, JTD, JHV, TGL, TM, LTF) have been involved in conceptualisation and writing of the manuscript. KTDF wrote the first draft and led the writing process. All authors have read and approved the final manuscript.

Acknowledgements

We thank the devoted clinical staff for their enthusiasm during the planning of the project as well as involved participants. We also thank all participating investigators in ATLAS4LAR including:

Bergen: Vibeke Bråthen Buljovcic, Jan Tore Daltveit, Karl Trygve Druckrey-Fiskaaen, Lars T. Fadnes (principal investigator), Trude Fondenes, Per Gundersen, Siv-Elin Leirvåg Carlsen, Mette Hegland Nordbotn, Maria Olsvold, Marianne Cook Pierron, Christine Sundal, and Jørn Henrik Vold.

Stavanger: Maren Borsheim Bergsaker, Eivin Dahl, Tone Lise Eielsen, Torhild Fiskå, Einar Furulund, Torgeir Gilje Lid, Eirik Holder, Tesfaye Madebo, Tine Selmer Cruickshank, and Ewa Joanna Wilk.

References

1. Gamble C, Krishan A, Stocken D, Lewis S, Juszczak E, Doré C, et al. Guidelines for the Content of Statistical Analysis Plans in Clinical Trials. Jama. 2017;318(23):2337-43.
2. Guydish J, Passalacqua E, Pagano A, Martinez C, Le T, Chun J, et al. An international systematic review of smoking prevalence in addiction treatment. Addiction. 2016;111(2):220-30.
3. Vlad C, Arnsten JH, Nahvi S. Achieving Smoking Cessation Among Persons with Opioid Use Disorder. CNS Drugs. 2020;34(4):367-87.
4. Morris CD, Garver-Apgar CE. Nicotine and Opioids: a Call for Co-treatment as the Standard of Care. J Behav Health Serv Res. 2020;47(4):601-13.
5. Fadnes LT, Aas CF, Vold JH, Leiva RA, Ohldieck C, Chalabianloo F, et al. Integrated treatment of hepatitis C virus infection among people who inject drugs: A multicenter randomized controlled trial (INTRO-HCV). PLoS Med. 2021;18(6):e1003653.
6. Druckrey-Fiskaaen KT, Furulund E, Daltveit JT, Vold JH, Lid TG, Madebo T, Fadnes LT. Integration of smoking cessation into standard treatment for patients receiving opioid agonist therapy who are smoking tobacco: protocol for a randomised controlled trial (ATLAS4LAR). Trials. 2022;23(1):663.
7. Sedgwick P. Spearman's rank correlation coefficient. Bmj. 2014;349:g7327.
8. European Medicines Agency CfHMP. Guideline for good clinical practice E6(R2). 6 ed. https://www.ema.europa.eu/en/documents/scientific-guideline/ich-e-6-r2-guideline-good-clinical-practice-step-5_en.pdf2018.
9. Development OfEC-oa. OECD Principles on Good Laboratory Practice. In: DIRECTORATE E, COMMITTEE CGAM, editors. https://www.oecd.org/officialdocuments/publicdisplaydocumentpdf/?cote=env/mc/chem(98)17&doclanguage=en1998.
