# Supplementary material for: Integrated Nicotine Replacement and Behavioral Support to Reduce Smoking in Opioid Agonist Therapy: A Randomized Clinical Trial
Source: JAMA Psychiatry. 2025 Feb 12;82(4):406–14. doi: 10.1001/jamapsychiatry.2024.4801 (PMC11822603; doi:10.1001/jamapsychiatry.2024.4801)
Supplement: Supplement 2. — eMethods 1. Details on Setting, Participants, and Interventions eMethods 2. Statistical Details eMethods 3. Details on Outcome and Measures eMethods 4. Sensitivity Analysis of Correlation of Self-Reported Cigarettes and Carbon Monoxide eTable 1. Demographics of Per Protocol Sample eTable 2. Participants’ Goals at the Start of the Trial (n = 135) eTable 3. Participants’ Estimation of the Time Needed to Achieve Smoking Reduction/Cessation Goal (n = 135) eTable 4. Participants’ Self-Reported Confidence in Success and Rating of Importance of Intervention at Baseline (n = 135) eTable 5. Overview of Study Visits Attended for Persons in the Intervention Group (n = 135) eTable 6. Adjusted Intention to Treat Analysis of Primary Outcome, With Imputation of Missing Variables (n = 259) eTable 7. Effect on Primary Outcome of Excluding Persons With Missing Values in the Primary Outcome eTable 8. Adjusted Intention to Treat Analysis of Primary Outcome, Without Imputation of Missing Variables (n = 200) eTable 9. Subgroup Analysis of the Primary Outcome Stratified by Baseline Variables eTable 10. Subgroup Analysis Assessing the Differential Effect of Various Subgroups on Primary Outcome (n = 259) eFigure 1. The Percentage of Study Visits Attended for Participants in the Intervention Arm eFigure 2. Timing of Drop-Out for Participants in the Intervention Arm eFigure 3. Scatter Plot of Self-Reported Number of Cigarettes and Carbon Monoxide Measurement eReferences [file jamapsychiatry-e244801-s002.pdf]

Supplemental Online Content

Druckrey-Fiskaaen KT, Madebo T, Daltveit JT, et al. Integrated nicotine replacement and behavioral support to reduce smoking in opioid agonist therapy: a randomized clinical trial. *JAMA Psychiatry*. Published online February 12, 2025.
doi:10.1001/jamapsychiatry.2024.4801

eMethods 1. Details on Setting, Participants, and Interventions
eMethods 2. Statistical Details
eMethods 3. Details on Outcome and Measures
eMethods 4. Sensitivity Analysis of Correlation of Self-Reported Cigarettes and Carbon Monoxide
eTable 1. Demographics of Per Protocol Sample
eTable 2. Participants' Goals at the Start of the Trial (n = 135)
eTable 3. Participants' Estimation of the Time Needed to Achieve Smoking Reduction/Cessation Goal (n = 135)
eTable 4. Participants' Self-Reported Confidence in Success and Rating of Importance of Intervention at Baseline (n = 135)
eTable 5. Overview of Study Visits Attended for Persons in the Intervention Group (n = 135)
eTable 6. Adjusted Intention to Treat Analysis of Primary Outcome, With Imputation of Missing Variables (n = 259)
eTable 7. Effect on Primary Outcome of Excluding Persons With Missing Values in the Primary Outcome
eTable 8. Adjusted Intention to Treat Analysis of Primary Outcome, Without Imputation of Missing Variables (n = 200)
eTable 9. Subgroup Analysis of the Primary Outcome Stratified by Baseline Variables
eTable 10. Subgroup Analysis Assessing the Differential Effect of Various Subgroups on Primary Outcome (n = 259)
eFigure 1. The Percentage of Study Visits Attended for Participants in the Intervention Arm
eFigure 2. Timing of Drop-Out for Participants in the Intervention Arm
eFigure 3. Scatter Plot of Self-Reported Number of Cigarettes and Carbon Monoxide Measurement
eReferences

This supplemental material has been provided by the authors to give readers additional information about their work.

© 2025 Druckrey-Fiskaaen KT et al. *JAMA Psychiatry*.

eMethods 1: Details on Setting, Participants, and Interventions

Description of outpatient clinics for opioid agonist treatment
The department of addiction medicine at Haukeland University Hospital in Bergen and the Department of substance abuse and addiction treatment at Stavanger University Hospital in Stavanger has implemented an integrated treatment and care model for patients enrolled in opioid agonist treatment (OAT). The clinics employ multidisciplinary teams including consultants, physicians specialized in addiction medicine, nurses, social workers, and in many cases, psychologists. Patients receive methadone, buprenorphine, or morphine sulphate for opioid dependency as directly observed medication and take-home medication. Depending on factor such as co-use of other drugs, mental and physical co-morbidities and stability in attendance patients are usually allowed between one and six take-home doses of the medication per week. During the COVID-19 pandemic medication was delivered at home by the OAT staff. In addition to OAT patients at the clinics each have been assigned at therapist (usually nurse or social worker) and are offered at least yearly health assessments by a physician and nurse. Persons in need of psychotherapy are referred to a psychologist at the OAT clinic. There is a close collaboration between the OAT-clinics and the patients primary care physicians, municipal social workers, and municipal activities for persons with substance dependence.

Details on participants, recruitment, and inclusion
We categorized participants in the study as "female" or "male" based on the classification in the medical records.

This study was conducted alongside two other studies evaluating the effect of fruit smoothie supplements and physical activity groups on mental health symptoms.[1,2] If eligible, participants were screened for all three studies and randomized for all three interventions and control groups simultaneously. Thus, participants randomized to the smoking reduction intervention did not necessarily have an intention to reduce or stop smoking.

Following randomization, seven participants (three from the intervention arm and four from the control arm) were excluded from the analysis: One person died shortly after randomization prior to study commencement. Five persons consented to study participation and were randomized but did not show up for the baseline study visit, thus provided no baseline data on the primary outcome, except screening data prior to inclusion. Because of the parallel enrolment in two other studies, as described above, one person who did not smoke was erroneously included in the study and was thus removed from analysis.

Detailed description of interventions
For both study arms, study nurses recruited participants, informed them, obtained written consent, assigned them to study arms, performed clinical interviews, completed study surveys, collected blood samples, and performed physical tests and spirometry. Study nurses completed these trial visits at baseline and 16 weeks. The primary outcome was assessed by study nurses in interviews.

Details on smoking interventions in the intervention arm
Participants in the intervention arm were at the trial study visit asked to choose a goal for their participation in the trial. The options were 'reduction', 'quit', or 'uncertain'. The were further asked to estimate how long the expected it would take to reach their goals. The participants were provided three alternatives: three months or less, three to six months or more than six months. Finally, the participants were asked to rate the importance of changing smoking habits and how certain they were that they could reach their goals. Both parameters were reported on an ordinal scale ranging from 0 (low) to 10 (high). The trial visits were conducted by the research nurses. The regular staff at the OAT clinics provided the interventions sessions offering nicotine replacement therapy (NRT) and behavioral support. During the 16 weeks of the trial participant engagement was flexible, allowing participants to stay in the trial for the whole duration even if they had missed one or more intervention sessions.

Details on smoking interventions in the standard treatment arm
At the time of the study, no smoking interventions were offered at OAT clinics. If a patient at an OAT clinic wanted to quit or reduce smoking, they were referred to their primary-care physicians. They were asked to buy NRT at pharmacies without any governmentally subsidized products available.

Reasons for ending the trial
The trial ended when the predefined number of participants to be included was reached.

© 2025 Druckrey-Fiskaaen KT et al. *JAMA Psychiatry*.

eMethods 2: Statistical Details

This trial used the Consolidated Standards of Reporting Trials (CONSORT) to guide the reporting of the findings.[3]

Statistical software used
We used Stata 18 (StataCorp, TX, USA) for the analyses and descriptive statistics.

Per protocol analysis
If data on the number of study visits attended were missing, participants were excluded from the per protocol analysis.

Details on post-hoc sample size calculations
In a post-hoc sample size calculation we estimated the number of participants needed to detect a difference in the proportion of participants achieving at least 50 % reduction in smoking. We assumed that 17% of participants in the control group achieved this. To detect a threshold of 35% of participants in the intervention group achieving the 50 % smoking reduction with 80% power, we would have needed 93 participants per group and 186 in total. With 90% power we would have needed 123 per group and 246 in total.

Details on sensitivity analyses
To assess the effect of missing data and it's handling on the results, we performed several sensitivity analyses: First, for the unadjusted analysis we tested the effect of handling missing data for the primary outcome as a complete case, i.e., excluding the participant from the analysis compared to setting missing outcome data equal to baseline. Secondly, for the adjusted analysis of the primary outcome we used multiple imputation to estimate values for missing data in the exposure variables. Spirometry results were missing for 50 participants, 15 persons lacked data on injection history, 5 persons had no data on year of smoking debut, and finally one person lacked data on cannabis use and packyears.

eMethods 3: Details on Outcome and Measures

The first author checked the range and completeness of the data reported by the study nurses prior to analysis, to ensure the data quality of the outcome data.

Carbon monoxide measurement

We measured carbon monoxide in the exhaled air using the Smokerlyzer® Pico (Bedfont® Scientific Ltd, Maidstone Kent, England) according to the manufacturer's instructions.

Definition of packyears

'Packyears' were defined as the daily number of cigarettes smoked multiplied by the number of years of smoking, divided by 20.

Details on assessment of tobacco amount

The average daily use of cigarettes was estimated by dividing the sum of cigarettes smoked during the past week by seven. If a participant smoked cannabis mixed with tobacco, they were asked to estimate the amount of tobacco (as a fraction of a cigarette) used when smoking cannabis. The number of cigarettes estimated from cannabis use was added to the estimate of the number of cigarettes smoked. We did not include use of e-cigarettes or other smokeless tobacco products such as snus in the estimates of daily cigarette use.

eMethods 4: Sensitivity Analysis of Correlation of Self-Reported Cigarettes and Carbon Monoxide

For both intervention arms the correlation of self-reported number of cigarettes smoked and carbon monoxide values at the end of the trial was similar among those reporting reducing the number of cigarettes smoked by at least one half: Intervention arm Spearman's rho = 0.417, p = 0.0132. Control arm Spearman's rho = 0.415, p 0.0693.

© 2025 Druckrey-Fiskaaen KT et al. *JAMA Psychiatry*.

eTable 1: Demographics of Per Protocol Sample

	Intervention (n=51)	Control (n=103)
Females n (%)	19 (37.3)	34 (33.0)
Age, mean (SD)	48.6 (10.6)	50.7 (10.3)
Age groups, n (%)		
<40	14 (27.5)	17 (16.5)
40-60	29 (56.9)	69 (67.0)
>60	8 (15.7)	17 (16.5)
BMI, mean (SD)	25.6 (5.1)	25.6 (6.4)
OAT medication[a], n (%)		
Methadone and others[b]	18 (35.3)	51 (49.5)
Buprenorphine	33 (64.7)	52 (50.5)
Stable living conditions[b], n (%)	49 (96.1)	96 (93.2)
Education, n (%)		
Not finished basic education[d]	1 (2.0)	5 (5.0)
Finished basic education[d]	23 (46.9)	47 (47.0)
High school and higher[e]	25 (51)	48 (48.0)
Social benefits as income, n (%)	50 (98.0)	102 (99.0)
Substance use[f]		
Opioids[g], n (%)		
none	46 (90.2)	82 (79.6)
< 3 times per week	3 (5.9)	17 (16.5)
> 3 times per week	2 (3.9)	4 (3.8)
Alcohol, n (%)		
none	26 (51.0)	49 (47.6)
< 3 times per week	19 (37.3)	41 (39.8)
> 3 times per week	6 (11.7)	13 (12.6)
Stimulants[h], n (%)		
none	39 (76.5)	72 (69.9)
< 3 times per week	11 (21.6)	21 (20.4)
> 3 times per week	1 (2.0)	10 (9.7)
Benzodiazepines, n (%)		
none	24 (47.1)	54 (52.4)
< 3 times per week	16 (31.4)	24 (23.3)
> 3 times per week	11 (21.5)	25 (24.3)
Cannabis, n (%)		
none	22 (43.1)	27 (26.2)
< 3 times per week	14 (27.5)	29 (28.2)
> 3 times per week	15 (29.4)	47 (45.7)
Tobacco, n (%)		
daily	48 (94.1)	99 (96.1)
Smoking debut (age), mean (SD)	14.4 (3.3)	14.1 (3.6)
Years of smoking, n (%)		
>15	50 (98.0)	98 (95.1)
Pack-years, mean (SD)	26.1 (19.1)	19.6 (14.2)
Cigarettes per day, mean (SD)	14.8 (8.5)	10.5 (6.6)
Carbon monoxide (ppm), mean (SD)	17.1 (7.6)	14.8 (7.9)
Probable COPD[i], n (%)	9 (20.0)	30 (34.1)
Injection tablets/ mixture, n (%)	5 (9.8)	11 (11.1)
Injection frequency, n (%)		
< 3 times per week	3 (60.0)	7 (63.6)
> 3 times per week	2 (40.0)	4 (36.4)

[a] Medication being used at baseline
[b] "Others" = Morphine sulfate formulations, in total 6 participants (3,9%)
[c] Living in an owned or rented home or being incarcerated was defined as a stable housing situation
[d] In Norway, the first ten school years are mandatory for all pupils.
[e] Grades 11-13.
[f] Self-reported frequency of substance use last 30 days prior to baseline assessment.
[g] Illegal opioids not part of the OAT program
[h] Amphetamines and cocaine
[i] At least one spirometry indicating FEV1/FVC ratio < Lower limit of normality[4]

© 2025 Druckrey-Fiskaaen KT et al. *JAMA Psychiatry*.

eTable 2: Participants' Goals at the Start of the Trial (n = 135)

Goal	N (%)
Reduce	32 (23.7)
Quit	57 (42.2)
Uncertain	9 (6.67)
Missing	37 (27.4)

eTable 3: Participants' Estimation of the Time Needed to Achieve Smoking Reduction/Cessation Goal (n = 135)

Timeframe	n (%)
≤ 3 months	28 (20.7)
3-6 months	45 (33.3)
> 6 months	16 (11.9)
Missing	46 (34.1)

eTable 4: Participants' Self-Reported Confidence in Success and Rating of Importance of Intervention at Baseline (n = 135)

	Mean	SD	Median	Range
Confidence[a]	6.70	2.45	7	10
Importance[a]	7.92	2.90	10	10

[a]Participants rated 0 as low and 10 as high

eTable 5: Overview of Study Visits Attended for Persons in the Intervention Group (n = 135)

Study visits attended	No. of participants (%)
0	42 (31.1)
1	4 (3.0)
2	4 (3.0)
3	4 (3.0)
4	5 (3.7)
5	2 (1.5)
6	5 (3.7)
7	14 (10.4)
8	9 (6.7)
9	14 (10.4)
10	5 (3.7)
11	4 (3.0)
12	9 (6.7)
13	3 (2.2)
14	7 (5.1)

© 2025 Druckrey-Fiskaaen KT et al. *JAMA Psychiatry*.

eTable 5: Overview of Study Visits Attended for Persons in the Intervention Group (n = 135) (continued)

Study visits attended	No. of participants (%)
15	3 (2.2)
16	1 (0.7)

eTable 6: Adjusted Intention to Treat Analysis of Primary Outcome, With Imputation of Missing Variables (n = 259)

Variable	Odds ratio	Std. error	95% CI	p-value
Intervention arm	1.82	0.58	0.97; 3.40	0.06
Age group	0.79	0.23	0.45; 1.39	0.42
Sex	0.75	0.26	0.39; 1.47	0.41
OAT medication	0.78	0.25	0.42; 1.46	0.44
COPD	0.54	0.22	0.24; 1.21	0.13
Injecting	0.44	0.24	0.15;1.26	0.13
Packyear	1.01	0.01	0.99; 1.04	0.19
Cannabis smoking	0.73	0.25	0.38; 1.42	0.35
Intercept	0.64	0.53	0.12; 3.29	0.59

eTable 7: Effect on Primary Outcome of Excluding Persons With Missing Values in the Primary Outcome

Outcome	Handling of missing data at end of trial	Events, n		Absolute difference between arms (n, 95 %CI)	Logistic Regression OR (95 % CI)	Adjusted logistic regression[a] OR (95% CI)
		Inter-vention	Control			
No. of smokers[b] at 16 weeks, ITT[c]	Equal to baseline[d]	134/135	119/124	3.3% (-0.39; 6.97)	0.17 (0.02;1.5) *p* = 0.117	0.22 (0.02; 2.64) p=0.23
	Person excluded[e]	112/113	98/103	4.0 % (0.04;8.37)	0.18 (0.02;1.52) *p* = 0.11	0.38 (0.03; 4.99) p=0.47
At least 50 % reduction number of cigarettes at 16 weeks, ITT[c]	Equal to baseline[d]	40/135	21/124	-12.7% (-23.01; -2.37)	2.07 (1.14; 3.75) p = 0.02	1.82 (0.97; 3.40) p= 0.06
	Person excluded[e]	40/113	21/103	-15.0% (-26.98;-3.03)	2.14 (1.16;3.96) *p* = 0.02	1.98 (1.03; 3.79) p=0.04

CI: Confidence interval; ITT: Intention to treat; OR: Odds ratio

[a] Adjusted for age group, sex, OAT medication, injection of mixture/ tablets, packyears, COPD and cannabis smoking at baseline. Missing exposure values were imputed, in total ?? imputations for ITT analysis.

[b] A person smoking at least one cigarette per day or seven cigarettes per week

[c] ITT = intention to treat population: Participants assessed according to randomization regardless of adherence to trial.

[d] If data on primary outcome is missing at 16 weeks, the results are set equal to baseline.

[e] If data on primary outcome is missing at 16 weeks the person is excluded from the analysis (complete case).

[f] PP = per protocol population: All participants who completed at least 50% of the trial visits

© 2025 Druckrey-Fiskaaen KT et al. *JAMA Psychiatry*.

eTable 8: Adjusted Intention to Treat Analysis of Primary Outcome, Without Imputation of Missing Variables (n = 200)

Variable	Odds ratio	Std. error	95% CI	p-value
Intervention arm	1.92	0.70	0.94; 3.94	0.07
Age group				
< 40 years	reference			
40 – 60 years	1.45	0.71	0.56; 3.78	0.44
> 60 years	0.69	0.50	0.17; 2.82	0.60
Male	reference			
Female	0.75	0.29	0.35; 1.59	0.45
Methadone and others	reference			
Buprenorphine	0.75	0.28	0.37; 1.55	0.44
No COPD	reference			
Probable COPD	0.41	0.18	0.17; 0.98	0.05
Not injecting	reference			
Injecting	0.41	0.24	0.13; 1.32	0.14
Packyear	1.01	0.01	0.99; 1.04	0.35
Smoking Cannabis	0.88	0.33	0.42; 1.85	0.74
Intercept	0.25	0.17	0.07; 0.91	0.04

© 2025 Druckrey-Fiskaaen KT et al. *JAMA Psychiatry*.

eTable 9: Subgroup Analysis of the Primary Outcome Stratified by Baseline Variables

	Odds ratio	Standard error	p-value	95% Confidence interval
Male (n = 179)				
Intervention arm	2.33	0.86	0.02	1.13; 4.79
Intercept	0.19	0.06	<0.001	0.11; 0.34
Female (n = 80)				
Intervention arm	1.57	0.86	0.41	0.54; 4.58
Intercept	0.22	0.09	<0.001	0.10; 0.51
< 40 years of age (n = 56)				
Intervention arm	0.69	0.44	0.56	0.20; 2.42
Intercept	0.38	0.18	0.04	0.15; 0.96
40-60 years of age (n = 167)				
Intervention arm	3.63	1.43	0.001	1.67; 7.90
Intercept	0.15	0.05	<0.001	0.08; 0.29
>60 years of age (n = 36)				
Intervention arm	0.80	0.68	0.78	0.15; 4.25
Intercept	0.27	0.15	0.02	0.09; 0.80
Methadone and others[a] (n = 122)				
Intervention arm	1.31	0.55	0.53	0.57; 3.00
Intercept	0.28	0.09	<0.001	0.15; 0.52
Buprenorphine (n = 137)				
Intervention arm	3.34	1.52	0.008	1.37; 8.15
Intercept	0.14	0.05	<0.001	0.07; 0.29
Injection of tablets or mixture[b] (n = 35)				
Intervention arm	0.44	0.44	0.41	0.06; 3.07
Intercept	0.25	0.16	0.032	0.07; 0.89
No injection of tablets or mixture[b] (n = 209)				
Intervention arm	2.60	0.87	0.004	1.35; 5.02
Intercept	0.20	0.05	<0.001	0.12; 0.33
Smoking < 10 cigarettes per day (n = 122)				
Intervention arm	0.85	0.40	0.73	0.34; 2.12
Intercept	0.25	0.08	<0.001	0.14; 0.46
Smoking ≥ 10 cigarettes per day (n = 137)				
Intervention arm	3.98	1.78	0.002	1.66; 9.55
Intercept	0.16	0.06	<0.001	0.07; 0.33
Smoking < 15 years (n = 12)				
Intervention arm	0.19	0.25	0.22	0.01; 2.66
Intercept	1.33	1.02	0.71	0.30; 5.96
Smoking ≥ 15 years (n = 246)				
Intervention arm	2.55	0.83	0.004	1.35; 4.82
Intercept	0.17	0.04	<0.001	0.10; 0.28

© 2025 Druckrey-Fiskaaen KT et al. *JAMA Psychiatry*.

eTable 9: Subgroup Analysis of the Primary Outcome Stratified by Baseline Variables (continued)

	Odds ratio	Standard error	p-value	95% Confidence interval
Packyears < 20 (n = 154)				
Intervention arm	1.41	0.57	0.40	0.63; 3.12
Intercept	0.21	0.06	<0.001	0.12; 0.38
Packyears ≥ 20 (n = 104)				
Intervention arm	3.42	1.62	0.010	1.35; 8.66
Intercept	0.20	0.08	<0.001	0.09; 0.42
No cannabis smoking (n = 66)				
Intervention arm	2.70	1.53	0.08	0.89; 8.18
Intercept	0.29	0.13	0.007	0.12; 0.71
Smoking Cannabis (n = 150)				
Intervention arm	1.83	0.70	0.11	0.87; 3.88
Intercept	0.25	0.07	<0.001	0.14; 0.43
No COPD (n = 148)				
Intervention arm	1.82	0.71	0.12	0.85; 3.89
Intercept	0.27	0.08	<0.001	0.14; 0.49
Probable COPD[c] (n = 61)				
Intervention arm	1.65	1.26	0.51	0.37; 7.34
Intercept	0.12	0.06	<0.001	0.04; 0.34

[a] "Others" = Morphine sulfate formulations
[b] Injection of Methadone mixture or Buprenorphine or Morphine sulfate tablets
[c] At least one spirometry indicating FEV1/FVC ratio < Lower limit of normality[4]

© 2025 Druckrey-Fiskaaen KT et al. *JAMA Psychiatry*.

eTable 10: Subgroup Analysis Assessing the Differential Effect of Various Subgroups on Primary Outcome (n = 259)

	Odds ratio	Standard error	p-value	95% Confidence interval
Subgroup sex.				
Intervention arm	2.33	0.86	0.02	1.33; 4.79
Male	reference			
Female	1.16	0.59	0.77	0.43; 3.16
Intervention arm# sex				
Male	reference			
Female	0.67	0.44	0.55	0.19; 2.45
Intercept	0.19	0.06	<0.001	0.11; 0.34
Age groups				
Intervention arm	0.69	0.44	0.56	0.20; 2.42
< 40	reference			
40-60	0.41	0.23	0.12	0.13; 1.26
>60	0.71	0.53	0.64	0.17; 3.03
Intervention arm# age group				
< 40	reference			
40-60	5.26	3.96	0.03	1.20; 22.97
>60	1.16	1.23	0.89	0.14; 9.34
Intercept	0.38	0.18	0.04	0.15; 0.96
OAT medication				
Intervention arm	1.31	0.55	0.53	0.57; 3.00
Methadone and others[a]	reference			
Buprenorphine	0.50	0.24	0.15	0.20; 1.30
Intervention arm# OAT medication				
Methadone and others[a]	reference			
Buprenorphine	2.56	1.59	0.13	0.76; 8.64
Intercept	0.28	0.09	<0.001	0.15; 0.52
Injection of tablets or mixture (15 imputations)				
Intervention arm	2.51	0.82	0.005	1.32; 4.77
No injection	reference			
Injection	1.21	0.84	0.78	0.31; 4.72
Intervention arm# Injection				
No injection	reference			
Injection	0.19	0.19	0.10	0.02; 1.41
Intercept	0.20	0.05	<0.001	0.12; 0.33
Smoking intensity (Packyears)				
Intervention arm	0.83	0.43	0.72	0.31; 2.27
Packyear[b]	0.98	0.02	0.36	0.95; 1.02
Intervention arm# packyear				
Packyear[b]	1.05	0.02	0.04	1.00; 1.09
Intercept	0.27	0.11	0.001	0.13; 0.58

© 2025 Druckrey-Fiskaaen KT et al. *JAMA Psychiatry*.

eTable 10: Subgroup Analysis Assessing the Differential Effect of Various Subgroups on Primary Outcome (n = 259) (continued)

	Odds ratio	Standard error	p-value	95% Confidence interval
Cigarettes smoked at baseline[c]				
Intervention arm	0.75	0.42	0.61	0.25; 2.24
Cigarettes at baseline	1.00	0.005	0.44	0.99; 1.01
Intervention arm# cigarettes	1.01	0.006	0.05	1.00; 1.02
Intercept	0.27	0.11	0.002	0.12; 0.61
Cannabis smoking				
Intervention arm	2.21	1.21	0.15	0.76; 6.44
No Cannabis smoking	reference			
Smoking Cannabis	0.81	0.43	0.70	0.28; 2.32
Intervention arm# Cannabis smoking				
No Cannabis smoking	reference			
Smoking Cannabis	0.85	0.56	0.80	0.23; 3.09
Intercept	0.24	0.11	0.002	0.10; 0.56
COPD (50 imputations)				
Intervention arm	1.86	0.67	0.08	0.92; 3.75
No COPD	Reference			
Probable COPD[d]	0.43	0.26	0.17	0.13; 1.42
Intervention arm# COPD				
No COPD	Reference			
Probable COPD[d]	0.97	0.83	0.97	0.18; 5.19
Intercept	0.26	0.08	<0.001	0.15; 0.46

[a] "Others" = Morphine sulfate formulations
[b] Per one unit increase in packyears
[c] Continuous, per one cigarette smoked at baseline
[d] At least one spirometry indicating FEV1/FVC ratio < Lower limit of normality[4]

© 2025 Druckrey-Fiskaaen KT et al. *JAMA Psychiatry*.

eFigure 1: The Percentage of Study Visits Attended for Participants in the Intervention Arm

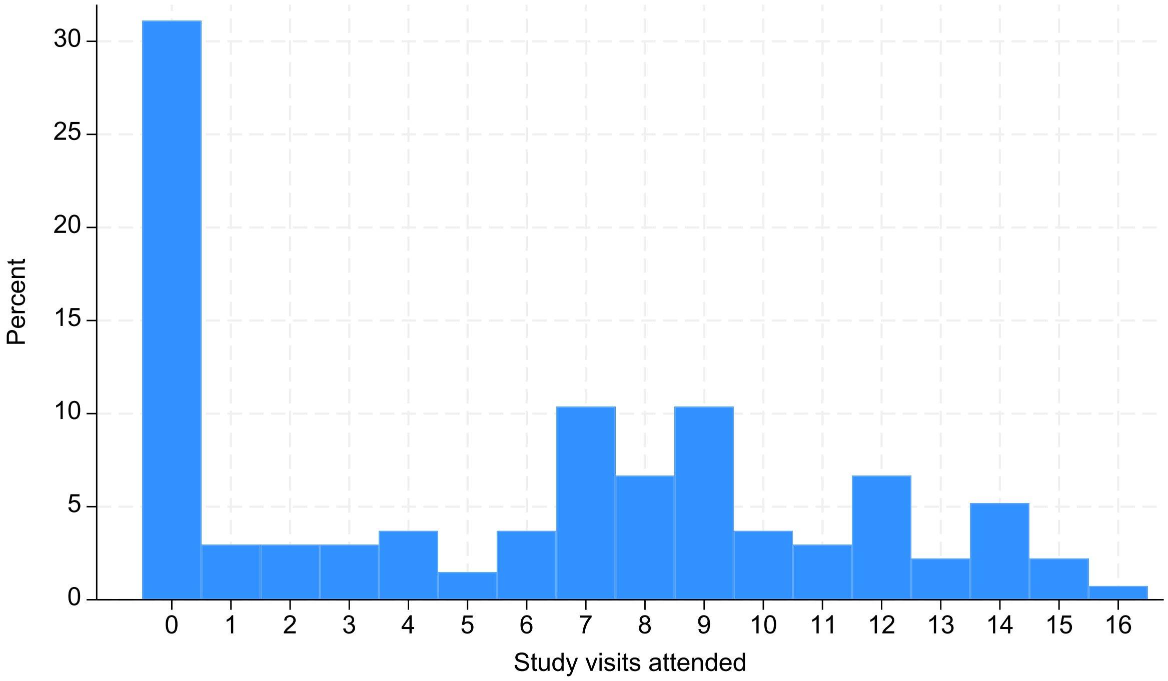

eFigure 2: Timing of Drop-Out for Participants in the Intervention Arm

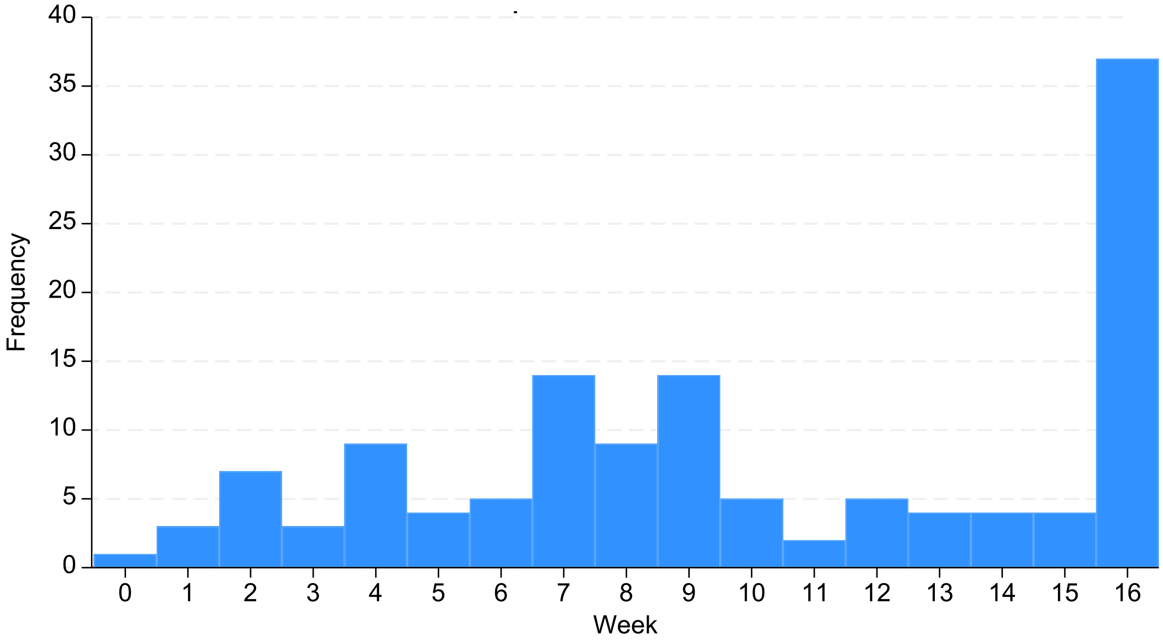

© 2025 Druckrey-Fiskaaen KT et al. *JAMA Psychiatry*.

eFigure 3: Scatter Plot of Self-Reported Number of Cigarettes and Carbon Monoxide Measurement

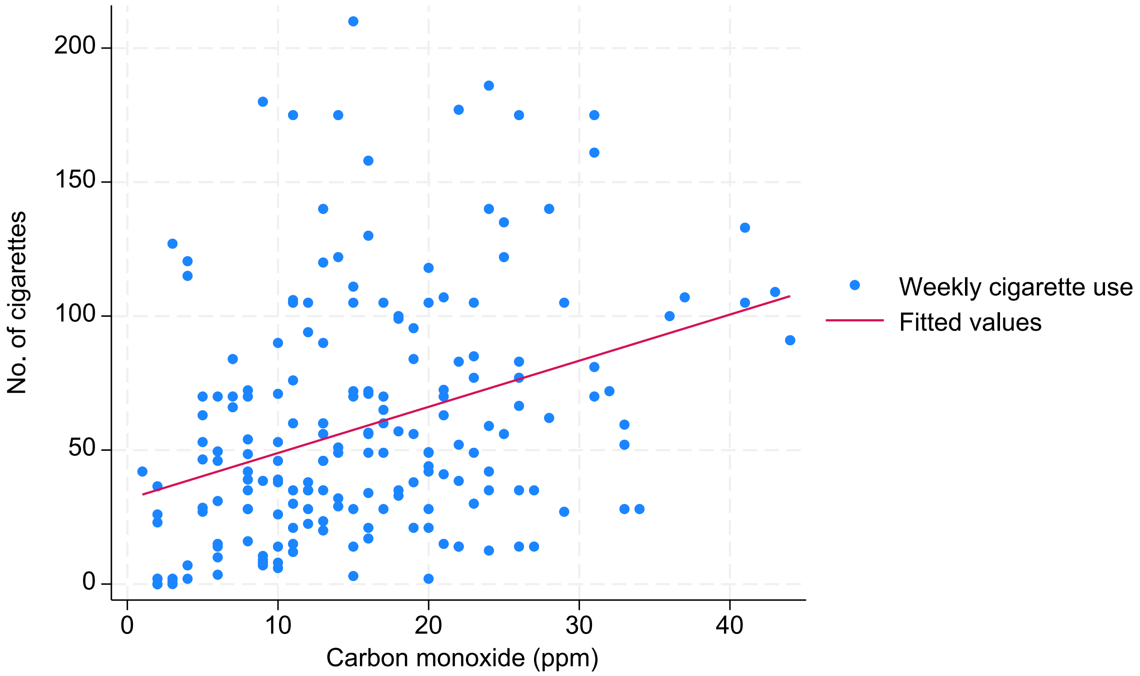

eReferences

1. Furulund E, Madebo T, Druckrey-Fiskaaen KT, et al. Integrated exercise program in opioid agonist therapy clinics and effect on psychological distress: study protocol for a randomized controlled trial (BAReAktiv). *Trials*. Feb 29 2024;25(1):155. doi:10.1186/s13063-024-07993-2

2. Fadnes LT, Furulund E, Druckrey-Fiskaaen KT, et al. Effect of fruit smoothie supplementation on psychological distress among people with substance use disorders receiving opioid agonist therapy: protocol for a randomised controlled trial (FruktBAR). *BMC Nutr*. Sep 3 2022;8(1):97. doi:10.1186/s40795-022-00582-z

3. Butcher NJ, Monsour A, Mew EJ, et al. Guidelines for Reporting Outcomes in Trial Reports: The CONSORT-Outcomes 2022 Extension. *Jama*. Dec 13 2022;328(22):2252-2264. doi:10.1001/jama.2022.21022

4. Agustí A, Celli BR, Criner GJ, et al. Global Initiative for Chronic Obstructive Lung Disease 2023 Report: GOLD Executive Summary. *Eur Respir J*. Apr 2023;61(4)doi:10.1183/13993003.00239-2023

© 2025 Druckrey-Fiskaaen KT et al. *JAMA Psychiatry*.
