# Supplementary material for: Integrated Nicotine Replacement and Behavioral Support to Reduce Smoking in Opioid Agonist Therapy: A Randomized Clinical Trial
Source: JAMA Psychiatry. 2025 Feb 12;82(4):406–14. doi: 10.1001/jamapsychiatry.2024.4801 (PMC11822603; doi:10.1001/jamapsychiatry.2024.4801)
Supplement: Supplement 3. — Data Sharing Statement [file jamapsychiatry-e244801-s003.pdf]

Data Sharing Statement

Druckrey-Fiskaaen. Integrated Nicotine Replacement and Behavioral Support to Reduce Smoking in Opioid Agonist Therapy. *JAMA Psychiatry*. Published February 12, 2025. doi:10.1001/jamapsychiatry.2024.4801

Data
Additional Information: ClinicalTrials.gov Identifier: NCT05290025
Data available: Yes
Data types: Deidentified participant data
How to access data: karl.fiskaaen@uib.no
When available: With publication

Supporting Documents
Document types: None

Additional Information
Who can access the data: Anyone requesting the data
Types of analyses: For the purpose of re-analyzing the study outcomes or for pooled analysis.
Mechanisms of data availability: The datasets generated will be made available with investigator support.
